# A Brain‐Targeting NIR‐II Ferroptosis System: Effective Visualization and Oncotherapy for Orthotopic Glioblastoma

**DOI:** 10.1002/advs.202206333

**Published:** 2023-03-03

**Authors:** Jing Zhang, Lulu Han, Haigang Wu, Yong Zhong, Ping Shangguan, Yisheng Liu, Mu He, Han Sun, Chenhui Song, Xin Wang, Yang Liu, Jiefei Wang, Lei Zheng, Bingyang Shi, Ben Zhong Tang

**Affiliations:** ^1^ Department of Laboratory Medicine Nanfang Hospital Southern Medical University 510515 Guangzhou China; ^2^ Henan‐Macquarie University Joint Centre for Biomedical Innovation School of Life Sciences Henan University 475004 Kaifeng China; ^3^ Key Laboratory for Special Functional Materials of Ministry of Education National & Local Joint Engineering Research Center for High‐efficiency Display and Lighting Technology School of Materials Science and Engineering Collaborative Innovation Center of Nano Functional Materials and Applications Henan University 475004 Kaifeng China; ^4^ Henan Key Laboratory of Brain Targeted Bio‐nanomedicine School of Life Sciences & School of Pharmacy Henan University 475004 Kaifeng China; ^5^ Macquarie Medical School Faculty of Medicine & Health Sciences Macquarie University Sydney NSW 2109 Australia; ^6^ School of Science and Engineering Shenzhen Institute of Aggregate Science and Technology The Chinese University of Hong Kong Shenzhen Guangdong 518172 China; ^7^ Department of Chemistry Hong Kong Branch of Chinese National Engineering Research Center for Tissue Restoration and Reconstruction and Institute for Advanced Study The Hong Kong University of Science and Technology Clear Water Bay Kowloon Hong Kong 999077 China

**Keywords:** aggregation‐induced emission, brain‐targeting, ferroptosis, glioma, near‐infrared imaging

## Abstract

Near‐infrared‐II (NIR‐II) ferroptosis activators offer promising potentials in in vivo theranostics of deep tumors, such as glioma. However, most cases are nonvisual iron‐based systems that are blind for in vivo precise theranostic study. Additionally, the iron species and their associated nonspecific activations might trigger undesired detrimental effects on normal cells. Considering gold (Au) is an essential cofactor for life and it can specifically bind to tumor cells, Au(I)‐based NIR‐II ferroptosis nanoparticles (TBTP‐Au NPs) for brain‐targeted orthotopic glioblastoma theranostics are innovatively constructed. It achieves the real‐time visual monitoring of both the BBB penetration and the glioblastoma targeting processes. Moreover, it is first validated that the released TBTP‐Au specifically activates the effective heme oxygenase‐1‐regulated ferroptosis of glioma cells to greatly extend the survival time of glioma‐bearing mice. This new ferroptosis mechanism based on Au(I) may open a new way for the fabrication of advanced and high‐specificity visual anticancer drugs for clinical trials.

## Graphical abstract

A near‐infrared (NIR) aggregation‐induced emission ferroptosis molecule (TBTP‐Au) is first designed by introducing an Au(I) active center. The self‐assembled TBTP‐Au nanoparticles with targeted modification display superior NIR imaging performance to monitor the blood–brain barrier penetration of nanoparticles. The controlled release of TBTP‐Au specifically activates the effective heme oxygenase‐1‐regulated ferroptosis to greatly prolong the survival of glioma‐bearing mice.

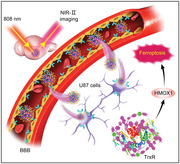

## 1 Introduction

Glioblastoma multiforme (GBM) is the most common and fatal malignant brain tumor,^[^ ^1^, ^2^, ^3^ ^]^ which faces the challenges of blood–brain barrier (BBB) and the lack of effective drugs,^[^ ^4^, ^5^, ^6^ ^]^ so there has been little progress in the treatment. Ferroptosis has emerged as a newly discovered iron‐dependent programmed cell death paradigm,^[^ ^7^, ^8^, ^9^ ^]^ and it is different from the common programmed cell death modalities including apoptosis, autophagy, necroptosis, and pyroptosis.^[^ ^10^ ^]^ Given neoplastic cells show higher sensitivity toward iron than those of the nonmalignant cells,^[^ ^11^, ^12^ ^]^ ferroptosis possesses great potential to kill malignancies. As documented, lipid peroxidation‐based ferroptosis generally can be classified into two types, i.e., canonical and noncanonical ferroptosis.^[^ ^13^ ^]^ Canonical ferroptosis involves the inactivation of glutathione peroxidase 4 (GPX4) which leads to uncontrolled and toxic lipid peroxidation.^[^ ^14^, ^15^ ^]^ Noncanonical ferroptosis includes excessive activation of heme oxygenase‐1 (HMOX1), where the increased labile iron pool could directly catalyze free radical formation via Fenton reaction and induce lipid peroxidation.^[^ ^16^, ^17^, ^18^ ^]^ Considering the short in vivo half‐lives and the incapability of penetrating BBB for current small‐molecule ferroptosis activators, such as sorafenib, sulfasalazine, and artemisinin,^[^ ^19^ ^]^ the iron‐based ferroptosis nanosystems^[^ ^20^, ^21^, ^22^ ^]^ and nonferrous (Mo and Cu)‐based nanomaterials^[^ ^23^, ^24^, ^25^ ^]^ were developed in recent years. However, these iron species and the associated nonspecific ferroptosis activation may trigger undesired detrimental effects,^[^ ^25^ ^]^ such as anaphylactic reactions in normal tissues.^[^ ^26^, ^27^, ^28^ ^]^ Moreover, these blind ferroptosis systems lack imaging capability and were unable to afford visual and precise theranostics in vivo. Therefore, constructing new nonferrous ferroptosis activators with visual imaging function in vivo is highly in demand.

Near‐infrared‐II (NIR‐II) fluorescence imaging has acquired considerable attention due to the advantages of deep tissue penetration and high imaging resolution,^[^ ^29^, ^30^, ^31^, ^32^ ^]^ which enables the real‐time observation of BBB penetration for nanoparticles.^[^ ^33^ ^]^ However, the existing inorganic NIR‐II probes have high risks of toxicity,^[^ ^34^ ^]^ such as upconverting nanoparticles (UCNP) and lead sulfide (PbS) quantum dots.^[^ ^35^, ^36^, ^37^ ^]^ In another aspect, organic dyes usually face severe aggregation‐caused quenching (ACQ) effects that may induce low imaging sensitivity.^[^ ^38^ ^]^ Aggregation‐induced emission luminogens (AIEgens), a type of special fluorophores that can overcome the above ACQ limitation,^[^ ^39^, ^40^, ^41^ ^]^ feature high biocompatibility and flexibly tailored photophysical performance and may be a promising option for the desired NIR‐II fluorophores. However, the short excitation/emission wavelength and lack of ferroptosis‐active centers make them still not been established for orthotopic glioma until now, which inevitably limit their applications. This reflects the huge challenge in molecule design and synthesis of NIR‐II ferroptosis activators including AIE systems.

**Figure 1:**
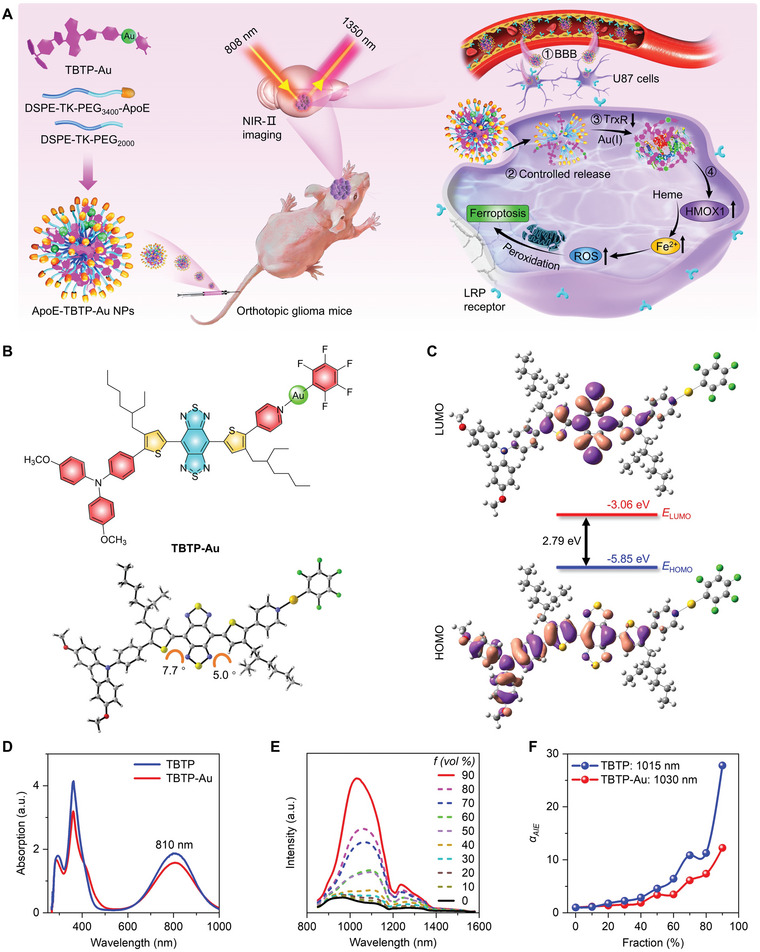
Structure and photophysical performance of TBTP‐Au. A) Schematic illustration of brain‐targeting ApoE‐TBTP‐Au NPs for ferroptosis in orthotopic glioma via the TrxR‐HMOX1 axis. B) Chemical structure and optimized S_0_ geometry of TBTP‐Au. C) Illustration of the frontier molecular orbitals (LUMO and HOMO) determined at the M062X‐D3/6‐31G(d,p) Lanl2DZ (Au) level of theory for TBTP‐Au. D) Absorption spectra of TBTP and TBTP‐Au in tetrahydrofuran (THF). E) Photoluminescence (PL) spectra (excitation: 808 nm) of TBTP‐Au in THF/*n*‐hexane mixtures with different fractions (*f*) of *n*‐hexane. F) Plot of PL peak intensity at 1015 nm of TBTP and 1030 nm of TBTP‐Au at various fraction values.

Based on the above points, we thus innovatively selected the biocompatible Au(I), an essential cofactor in humans, as the nonferrous active center to design a NIR‐II ferroptosis activator (TBTP‐Au) by incorporating an AIE‐active molecule backbone. The pertinent results turned out that real‐time NIR‐II imaging could be realized, which efficiently monitored the BBB penetration and targeting process of apolipoprotein E (ApoE) peptide^[^ ^42^ ^]^ modified TBTP‐Au nanoparticles (ApoE‐TBTP‐Au NPs). Additionally, triggered by the tumor microenvironment,^[^ ^43^ ^]^ the controlled release of TBTP‐Au from the reactive oxygen species (ROS)‐responsive nanosystem could initiate effective death of glioma cells through selectively binding to the overexpressed thioredoxin reductase (TrxR),^[^ ^44^, ^45^ ^]^ and specifically activate HMOX1‐regulated ferroptosis pathway (**Figure** **1**A). Impressively, this finding is completely distinct from the current reports on the mechanisms of nonspecific noncanonical ferroptosis and the known Au‐induced autophagy and apoptosis.^[^ ^46^, ^47^ ^]^ This study demonstrates the first example of a high‐specificity NIR‐II ferroptosis activator against glioma. Moreover, the present strategy may inspire more discoveries and be helpful for the development of advanced visual anticancer drugs.

## 2 Results and Discussion

### 2.1 Molecular Design, Synthesis, and Photophysical Properties of TBTP‐Au

Regarding the design of AIE core, we selected a unique donor (D)‐*π*‐acceptor (A)‐*π*‐A type of twisted structure (TBTP), where benzo[1,2‐c:4,5‐c’]bis([1,2,5]thiadiazole) (BBTD) was a common core acted as the electron acceptor for NIR‐II molecule, thiophene ring was *π*‐conjugation part and triphenylamine (TPA) played as the electron donor (Figure 1B). As we mentioned above, Au(I) is a biocompatible active center of clinically approved antirheumatic drug (auranofin),^[^ ^45^ ^]^ which has the potential to trigger ferroptosis via specifically inhibiting the highly expressed TrxR in tumor cells. So we further engaged the Au(I) unit into the AIE‐active TBTP core to obtain the target molecule, TBTP‐Au, and further evaluated its potential both in NIR‐II imaging in vivo and ferroptosis activation. To this end, the target molecule TBTP‐Au was synthesized via the well‐established synthetic route (Figure S1, Supporting Information). To validate the contribution of Au(I), the control molecule, TBTP, was simultaneously studied. The structures of all intermediates were confirmed by nuclear magnetic resonance (NMR) and high‐resolution mass spectrum (HRMS) (Figures S2–S11, Supporting Information).

Next, the photophysical performance of TBTP‐Au was evaluated. Firstly, the preliminary theoretical calculation was performed. The electron cloud of the lowest unoccupied molecular orbital (LUMO) was dominantly distributed on the central BBTD unit, and the highest occupied molecular orbital (HOMO) was delocalized across the whole conjugated backbone of TPA and two thiophene rings, which generated a bandgap of 2.79 eV (Figure 1C). Thus, this typical charge separation character in the donor–acceptor structure was favorable for the redshift of both the absorption and emission spectra compared with the electron‐withdrawing BBTD core.^[^ ^33^ ^]^ Therefore, both TBTP and TBTP‐Au exhibited a long absorption to ≈1000 nm (Figure 1D). Further photoluminescence (PL) experiments demonstrated the typical AIE properties of both TBTP and TBTP‐Au. As depicted in Figure 1E,F, a gradually intensified PL profile with the maximum peak at 1030 nm for TBTP‐Au was observed when increasing the fraction of insoluble solvent (*n*‐hexane). Comparatively, TBTP showed a blue‐shifted peak at 1015 nm in the aggregate state (Figure S12, Supporting Information). Given the long NIR‐II luminescent property, the developed TBTP‐Au has the potential to act as a visual platform for in vivo deep‐penetration bioimaging.

### 2.2 Physical, Optical Performance, and Tumor Selective Inhibition

**Figure 2:**
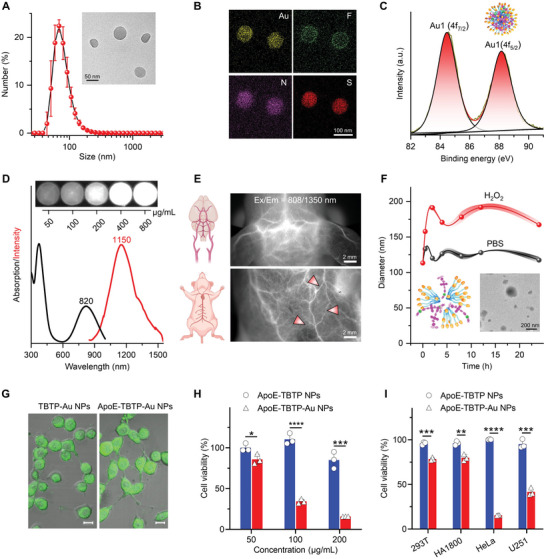
Photophysical performance and in vitro anticancer activity of TBTP‐Au NPs. A) Size distribution graph and corresponding TEM image of TBTP‐Au NPs. B) Element mapping images of Au, F, N, and S in TBTP‐Au NPs by scanning transmission electron microscopy (STEM). C) XPS spectra of Au in TBTP‐Au NPs. D) Absorption and fluorescence spectra (excitation: 808 nm) of TBTP‐Au NPs. The inset shows NIR imaging at various concentrations of TBTP‐Au NPs aqueous solution using a 1350 nm filter. E) In vivo NIR imaging at 1350 nm of cerebrovascular and body blood vessels. F) Changes in diameter of TBTP‐Au NPs in PBS and H_2_O_2_ solution during a 24 h of incubation. The inserted TEM image shows degraded TBTP‐Au NPs incubated with H_2_O_2_ for 6 h. G) Confocal images of U87 cells treated with two NPs for 2 h. Scale bars: 10 µm. H) Cell toxicity of two NPs with various concentrations to U87 cells (*n* = 3). I) Toxicity assessment of two NPs to various cell lines (*n* = 3). Data are presented as mean ± standard deviation (SD). *p < 0.05, **p < 0.01, ***p < 0.001, and ****p < 0.0001. Unless otherwise stated, all concentrations of nanoparticles were 100 µg mL^−1^ and U87‐luciferase cells (U87‐Luc) were used in whole‐cell assays.

Furthermore, the TBTP‐Au molecule was self‐assembled into 126.5 nm of sphere‐like smart nanoparticles (NPs) (**Figure** **2**A). The results of element mapping demonstrated the homogeneous distribution of Au in nanoparticles, and the two characteristic peaks at 84.5 eV (Au4f_7/2_) and 88.2 eV (Au4f_5/2_) in X‐ray photoelectron spectroscopy (XPS) spectra (Figure 2B,C) further indicated Au(I) not Au(III) or zero states.^[^ ^48^ ^]^ The NPs also showed superior stability over a 5‐day incubation in various media (Figure S13, Supporting Information). The maximum peaks of the absorption and PL spectra for TBTP‐Au NPs red‐shifted to 820 and 1150 nm, respectively (Figure 2D), which was well consistent with the results of strong concentration‐dependent NIR‐II brightness under 1350 nm (image inset; other wavelengths are shown in Figure S14, Supporting Information). Furthermore, bright cerebrovascular and blood vessel frames under a 1350 nm filter were observed (Figure 2E) and preliminarily confirmed the prominent NIR‐II imaging ability of TBTP‐Au NPs.

Prior to in vitro tumor selective inhibition assays, we first verified the ROS‐responsive release triggered by hydrogen peroxide (H_2_O_2_) and the tumor‐targeting capability of NPs. As shown in Figure 2F (inset image), the increased diameter and transmission electron microscopy (TEM) image confirmed the successful release. The results of Fourier‐transform infrared spectroscopy (FTIR) further proved the successful modification of ApoE (Figure S15, Supporting Information). Assisted by the green fluorescence ranging from 500 to 600 nm of NPs (Figure S16, Supporting Information), the confocal images of U87 cells incubated with target NPs showed higher uptake in the cytoplasm than that of nontarget NPs (Figure 2G). The optical value at 820 nm also verified it and revealed the maximum at 8 h (Figure S17, Supporting Information). The result of 3D tumor spheroids indicated ApoE‐TBTP‐Au NPs have superior tumor‐penetrating and targeting capability (Figure S18, Supporting Information). Additionally, in sharp contrast to the neglectable toxicity of Au‐free ApoE‐TBTP NPs, the released TBTP‐Au molecules from ApoE‐TBTP‐Au NPs triggered dramatic concentration‐dependent toxicity in U87 cells (Figure 2H). Furthermore, it exhibited significant selective damage to tumor cells (HeLa and U251 cells) compared with 293T and HA1800 cells (Figure 2I). The results of flow cytometry also presented a higher death rate of U87 cells (70.47%) caused by ApoE‐TBTP‐Au NPs than that of PBS (8.78%) and Au‐free NPs (7.77%, Figure S19, Supporting Information). These results indicated that ApoE‐TBTP‐Au NPs could play as specific and effective NIR‐II nanotheranostic for glioma cells, where Au(I) acted as the active center.

### 2.3 Unveiling of TrxR/HMOX1 Ferroptosis Axis

**Figure 3:**
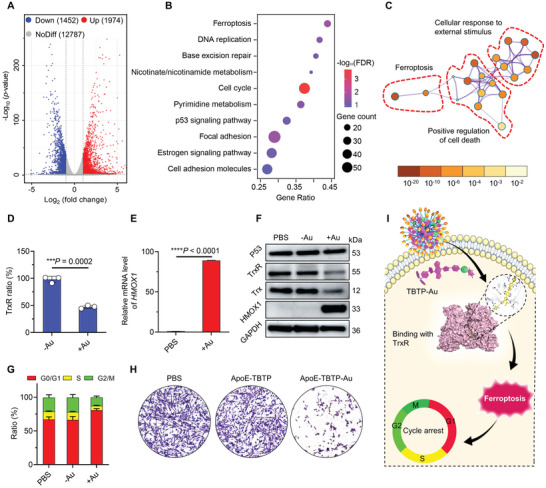
TrxR/HMOX1 ferroptosis and cell cycle arrest induced by ApoE‐TBTP‐Au NPs. A) Volcano plot of all identified genes in U87 cells treated with ApoE‐TBTP‐Au NPs. Threshold value to consider as significance (FDR *q* value < 0.05 and log_2_|Fold Change| ≥ 1.0). B) Bubble diagram of KEGG enrichment analysis of the up‐regulated DEGs after treatment with ApoE‐TBTP‐Au NPs. FDR and gene count are represented by bubble color and size, respectively. FDR *q* value of all presented terms is less than 0.05. C) Pathway enrichment analysis of the up‐regulated DEGs using Metascape. Enrichment significance of gene or gene clusters was presented as bubble color, which is less than 0.05. Dots occurred by red circle were attributed to same pathway. D) In vitro TrxR levels in U87 cells treated with various NPs for 24 h (*n* = 3). E) Relative mRNA expression of *HMOX1* in U87 cells treated with NPs and PBS (*n* = 3). F) Western blot of TrxR, Trx, HMOX1, and P53 proteins in U87 cells after treatments for 24 h. G) Cell cycle analysis and quantification of U87 cells with various treatments for 24 h by flow cytometry (*n* = 3). H) Representative crystal violet staining of U87 cells after various treatments for 24 h. I) Schematic illustration of ApoE‐TBTP‐Au NPs induced ferroptosis following cell cycle arrest after binding with TrxR. ApoE‐TBTP NPs and ApoE‐TBTP‐Au NPs were respectively termed “−Au” and “+Au”. Data are presented as mean ± standard deviation (SD). ***p < 0.001, ****p < 0.0001.

To unveil the underlying molecular mechanism of ApoE‐TBTP‐Au NPs against glioma cells, we conducted RNA sequencing to investigate the gene expression profile of U87 cells after various treatments. Firstly, all gene expressed levels were performed (Figure S20, Supporting Information), and we identified 3426 differentially expressed genes (DEGs) for the ApoE‐TBTP‐Au NPs post‐treated group, including 1452 down‐regulated DEGs and 1974 up‐regulated DEGs (**Figure** **3**A). To pull out the potential anticancer pathways from these DEGs, Kyoto Encyclopedia of Genes and Genomes (KEGG) enrichment analysis were performed. The term plot results presented in Figure 3B and Figure S21 (Supporting Information) clearly showed that the ferroptosis‐related pathway was significantly enriched, implying the critical role in inducing cell death by NPs treatment. Further clustering enrichment analysis and Gene Set Enrichment Analysis (GSEA, Figure S22A, Supporting Information) corroborated that the ferroptosis response was coupled with positive regulation of external stimulus and cell death (Figure 3C). To identify the hub genes in modulating ferroptosis pathways during NPs treatment, we performed the protein–protein interaction (PPI) network analysis, and we discovered some HMOX1‐centered markers that had strong regulatory effects on ferroptosis (Figure S22B, Supporting Information). Similar to TrxR, HMOX1 was also a redox regulator. Therefore, we speculated a TrxR inhibition‐induced HMOX‐1 activation as the Au(I) could specifically inhibit the activity of TrxR.^[^ ^44^ ^]^ To verify this hypothesis, the level of cellular TrxR and HMOX1 were determined using the TrxR assay kit and the quantitative polymerase chain reaction (qRT‐PCR), respectively. We observed an obvious decrease of TrxR (Figure 3D) and substantial up‐regulation (89.1‐fold) of *HMOX1* than the other DEGs (Figure 3E and Figure S23, Supporting Information). Likewise, the result of immunoblots confirmed the same conclusion (Figure 3F). Taken together, the above results convincingly pointed out that TBTP‐Au selectively activated the HMOX1‐regulated noncanonical ferroptosis but not autophagy and apoptosis.

**Figure 4:**
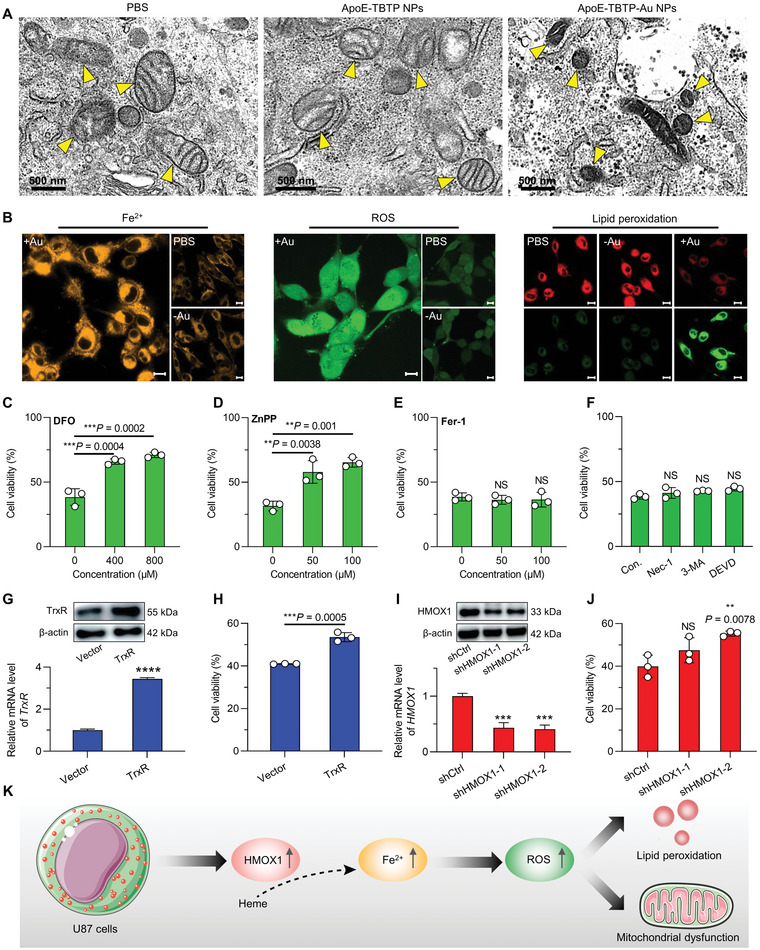
In vitro analyses of ferroptosis phenotype. A) Ferroptosis‐induced morphological change by Bio‐TEM observation in mitochondria from U87 cells after the treatment for 24 h. B) Confocal images of labile iron (Fe^2+^), ROS and lipid peroxidation in U87 cells stained with FerroOrange, DCFH‐DA, and BODIPY probes, respectively. Scale bars: 10 µm. C–F) Cell viability of U87 cells co‐incubated with ApoE‐TBTP‐Au NPs (100 µg mL^−1^) and different concentrations of ferroptosis inhibitors deferoxamine (DFO (C)), Zinc protoporphyrin (ZnPP (D)), Ferrostatin‐1 (Fer‐1 (E)) and other specific inhibitors (F). The control group was ApoE‐TBTP‐Au NPs treatment without any inhibitor in (F), which was the same as the 0 groups in (C), (D), and (E). G) *TrxR* expression by qRT‐PCR and immunoblot in TrxR over‐expressing U87 cells (*n* = 3). H) Corresponding reversal of cell viability in TrxR over‐expressing U87 cells after treatment with ApoE‐TBTP‐Au NPs for 24 h (*n* = 3). I) *HMOX1* expression by qRT‐PCR and immunoblot in HMOX1 down‐regulating U87 cells. J) The corresponding cell viability treated with ApoE‐TBTP‐Au NPs for 24 h (*n* = 3). K) Schematic illustration of the ferroptosis process in U87 cells. Unless stated, all concentrations of two NPs were 100 µg mL^−1^. ApoE‐TBTP NPs and ApoE‐TBTP‐Au NPs were respectively termed “−Au” and “+Au.” Data are presented as mean ± SD, **p < 0.01, ***p < 0.001, and ****p < 0.0001, NS, no significance.

Furthermore, we verified the detailed process of HMOX1‐mediated ferroptosis characterized by lipid peroxidation to cell death. Firstly, the cell cycles that reflected cell activity were assessed on the U87 cells. The ApoE‐TBTP‐Au NPs group indicated an S phase cell‐cycle arrest (6.57%) compared with the PBS (11.86%) or Au‐free NPs (11.88%) group, revealing a DNA replication inhibition, which might be induced by the lipid peroxidation stress as shown in Figure 3G. The crystal violet staining was used to assess the therapeutic efficiency, and the result indicated an obvious cell proliferation inhibition after the treatment of ApoE‐TBTP‐Au NPs for 24 h (Figure 3H). In addition, as shown in Figure 3I, the ApoE‐TBTP‐Au NPs efficiently induced the death of U87 cells through ferroptosis, accompanied by cycle arrest and suppression of the growth. Besides the cellular proliferation inhibition, it simultaneously damaged the cell membranes and organelles, which might further induce cell death. As depicted in **Figure** **4**A and Figure S24 (Supporting Information), drastic morphological damage of the cell membranes, decreased volume of the mitochondrial cristae and obvious shrinkage of the mitochondrial profile as well as condensation of the mitochondrial membranes were observed in Bio‐TEM images, indicating typical ferroptosis‐involved cell death. Moreover, the lipid peroxidation was triggered by HMOX1 that catalyzed the degradation of heme into labile iron and increased the ROS level via Fenton reaction. In addition to the above morphological changes, these three intermediates were also tested using three specific probes, respectively, i.e., FerroOrange for labile Fe^2+^ detection, 2′,7′‐dichlorofluorescein diacetate (DCFH‐DA) for ROS detection, and commercial BODIPY probe for lipid peroxidation. As clearly depicted in Figure 4B, bright fluorescence for the ApoE‐TBTP‐Au NPs treated groups were all observed under the signaling of these three different probes, strongly suggesting the lipid peroxidation‐associated cell death pathway. Moreover, both the results of electron paramagnetic resonance (EPR) and 3,3′,5,5′‐tetramethylbenzidine (TMB) screening indicated that ApoE‐TBTP‐Au NPs were unable to generate radical (Figure S25, Supporting Information), and the radical in the ferroptosis was generated from the HMOX1‐regulated endogenous Fe^2+^, but not from Au(I).

Apart from the above validation, a series of reverse cellular investigations of various inhibitors on cell viability were also assessed to confirm the ferroptosis regulation axis. As presented in Figure 4C,D, the viability of U87 cells treated by a labile iron chelator (DFO)^[^ ^49^ ^]^ or the HMOX1 inhibitor (ZnPP)^[^ ^50^ ^]^ exhibited an obvious increase. Whereas the treatment of ferroptosis inhibitor (Fer‐1) failed to improve the cell viability (Figure 4E) because of Fer‐1 was a common inhibitor for GPX4‐mediated ferroptosis.^[^ ^51^ ^]^ In addition, the necrosis inhibitor (Necrostatin‐1, Nec‐1), autophagy inhibitor (3‐Methyladenine, 3‐MA), and apoptosis inhibitor (Z‐DEVD‐FMK, termed as DEVD) were also utilized to further assess the cell death modality.^[^ ^52^ ^]^ However, these all failed to improve the cell viability (Figure 4F), suggesting that the inhibition pathway of tumor cells was independent of the above three modalities. Subsequently, the effects of the ferroptosis targets (TrxR and HMOX1) on cell viability were assessed using TrxR‐overexpressed and HMOX1 knocking down U87 cells, respectively. In the TrxR‐overexpressed cells verified by the mRNA and protein levels (Figure 4G), the cell viability of the ApoE‐TBTP‐Au NPs treated group showed more obvious improvement than that of the control one (Figure 4H). Likewise, in the HMOX1 knocking down cells (Figure 4I), a significantly improved cell viability was also observed as shown in Figure 4J. These results consistently demonstrated that ApoE‐TBTP‐Au NPs could act as an efficient ferroptosis activator and effectively exert antitumor activity via the TrxR‐HMOX1 axis, which caused lipid peroxidation and mitochondrial dysfunction and further resulted in cell death (Figure 4K).

### 2.4 In Vivo Theranostic Effects of ApoE‐TBTP‐Au NPs

**Figure 5:**
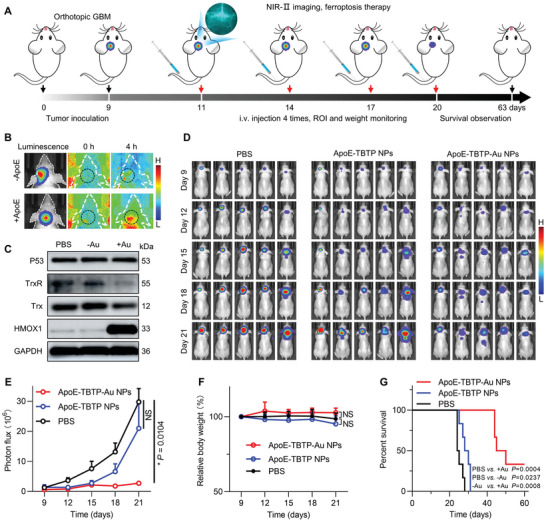
Theranostic effects of ApoE‐TBTP‐Au NPs on orthotopic glioma model. A) Timeline of theranostic treatment in mice bearing orthotopic U87‐Luc tumor. B) Real‐time NIR imaging of orthotopic glioma‐bearing mice before and at 4 h post injection of ApoE‐TBTP‐Au NPs (+ApoE) and TBTP‐Au NPs (−ApoE) (dosage: 10 mg kg^−1^). C) Expression of ferroptosis‐related proteins by Western blot in ex vivo tumors after finishing treatment. D) In vivo bioluminescence imaging of mice at various days post *i.v*. injection with PBS, ApoE‐TBTP NPs, and ApoE‐TBTP‐Au NPs, respectively (*n* = 5). The images were captured on the next day after injection except that on day 9. E) Quantified luminescence values in tumors (*n* = 5). Data are presented as mean with standard error of the mean (SEM). F) The corresponding weight changes of mice (*n* = 6) undergoing treatment. G) Survival graph of the tumor‐bearing mice (*n* = 6). NS, no significance, *p < 0.05. ApoE‐TBTP NPs and ApoE‐TBTP‐Au NPs were respectively termed “−Au” and “+Au.”

Considering the superior anticancer property of ApoE‐TBTP‐Au NPs at the cellular level, we then evaluated the in vivo therapeutic efficacy in the orthotopic GBM mouse model according to the predesigned protocol (**Figure** **5**A). Prior to the therapy, the biocompatibility of the NPs was first determined by assessing the effects on hemolysis and morphology of red blood cells (Figure S26, Supporting Information), as well as routine blood parameters and blood biochemistry (Figure S27, Supporting Information) in healthy BALB/c mice. The low hemolysis ratio (<5%), intact morphology of red blood cells as well as normal blood parameters and biochemistry together showed the excellent biocompatibility of ApoE‐TBTP‐Au NPs. The in vivo pharmacokinetic study of mice after *i.v*. injection of ApoE‐TBTP‐Au NPs (dosage: 10 mg kg^−1^) showed a similar circulation time (*t*_1/2,_ *_*β*_* = 3.3 h) to that of the treatment with nontargeting TBTP‐Au NPs (*t*_1/2,_ *_*β*_* = 3.2 h) (Figure S28, Supporting Information). The results of NIR‐II imaging applied to monitor the tumor accumulation of NPs (Figure 5B) together with the fluorescence intensity quantification (Figure S29, Supporting Information) indicated that ApoE‐TBTP‐Au NPs showed much higher BBB‐penetrating and tumor‐accumulating abilities compared with that of TBTP‐Au NPs group. Simultaneously, the ApoE‐TBTP‐Au NPs mainly accumulated in the liver and kidney as determined by the absorption of tissue homogenates at 835 nm (Figure S30, Supporting Information). After a therapeutic process performed by *i.v*. injection of ApoE‐TBTP‐Au NPs every three days for four cycles, the expression of TrxR, Trx, HMOX1, and P53 proteins for the harvested brain tumor tissues showed evident changes, which were well consistent with the results observed at the cellular level (Figure 5C). The tumor growth was also monitored by bioluminescence images (Figure 5D) and evaluated by the quantified luminescent intensity (Figure 5E). The group treated with ApoE‐TBTP‐Au NPs showed a strong tumor inhibition effect with negligible change in body weight (Figure 5F). More impressively, the treatment of ApoE‐TBTP‐Au NPs could significantly improve the survival period to more than 60 days (Figure 5G), in sharp contrast to the 28 days of the PBS control group and the 31 days of the non‐Au group. Furthermore, biosafety assessment by hematoxylin‐eosin (H&E) staining indicated no obvious physiological abnormalities (Figure S31, Supporting Information). Therefore, the fabricated biocompatible ApoE‐TBTP‐Au NPs as a powerful NIR‐II ferroptosis activator could be applied in NIR‐II imaging‐guided theranostics of glioma.

## 3 Conclusion

The high‐specificity anticancer drugs can minimize the toxicity to normal cells and maximize therapeutic efficiency. To address the challenges of iron toxicity, nonspecific activation and non‐visualization of the current iron‐based systems, we first synthesized an Au(I)‐based NIR‐II ferroptosis activator (TBTP‐Au) with AIE property by integrating Au(I) unit and AIE backbone. Then, TBTP‐Au was self‐assembled into the brain‐targeting nanodrug via ROS‐responsive template and ApoE modification. As a result, the fabricated nanodrug achieved real‐time NIR‐II imaging to monitor the BBB penetration and the targeting to glioma cells. It was well demonstrated that the higher ROS gradient in the glioma microenvironment stimulated the release of TBTP‐Au from the NPs, and Au(I) selectively bound with the over‐expressed TrxR and triggered the specific HMOX1‐regulated noncanonical ferroptosis activation of glioma cells, which induced notable tumoral suppression and the extension of the survival time. The proposed fabrication and modulation rules in this work would give favorable impetus for the development of new NIR‐II ferroptosis activators. Moreover, the Au‐based nanoparticles also have great potential as powerful photothermal agents or radiosensitizers to boost anticancer efficacy, which may provide a toolbox for developing more exciting metal‐engaged drugs with visualization capacity and high therapeutic specificity, and further inspire researchers to explore more unknown mechanisms.

## Conflict of Interest

The authors declare no conflict of interest.

## Supporting information

Supporting Information

## Data Availability

The data that support the findings of this study are available from the corresponding author upon reasonable request.
