# Supplementary material for: A Brain‐Targeting NIR‐II Ferroptosis System: Effective Visualization and Oncotherapy for Orthotopic Glioblastoma
Source: Adv Sci (Weinh). 2023 Mar 3;10(13):2206333. doi: 10.1002/advs.202206333 (PMC10161027; doi:10.1002/advs.202206333)
Supplement: Supplementary file 1 — Supporting Information [file ADVS-10-2206333-s001.pdf]

ADVANCED SCIENCE
Open Access

Supporting Information

for *Adv. Sci.*, DOI 10.1002/advs.202206333

A Brain-Targeting NIR-II Ferroptosis System: Effective Visualization and Oncotherapy for Orthotopic Glioblastoma

Jing Zhang, Lulu Han, Haigang Wu, Yong Zhong, Ping Shangguan, Yisheng Liu, Mu He, Han Sun, Chenhui Song, Xin Wang, Yang Liu, Jiefei Wang, Lei Zheng*, Bingyang Shi* and Ben Zhong Tang**

Supporting Information

A Brain-Targeting NIR-II Ferroptosis System: Effective Visualization and Oncotherapy for Orthotopic Glioblastoma

Jing Zhang, Lulu Han, Haigang Wu, Yong Zhong, Ping Shangguan, Yisheng Liu, Mu He, Han Sun, Chenhui Song, Xin Wang, Yang Liu, Jiefei Wang, Lei Zheng,* Bingyang Shi,* and Ben Zhong Tang**

Experimental section

Chemicals: All chemicals and biological reagents were purchased from known suppliers and used without further purification. DSPE-thioketal (TK)-PEG_{2000}-OCH_3 and DSPE-TK-PEG_{3400}-Mal were obtained from Xi'an ruixi Biological Technology Co., Ltd. (Xi'an, China). $ApoE(159\text{-}167)_2$ with a sequence $(LRKLRKRLL)_2C$ and cysteine on C-terminal (ApoE-SH) was purchased from ChinaPeptides Co., Ltd. (Shanghai, China). Cytotoxicity assay kit (CCK-8) and Micro Oxidized Thioredoxin Reductase (TrxR) Assay Kit were bought from Solarbio Science & Technology Co., Ltd. (Beijing, China). 2′, 7′-dichlorodihydrofluorescein diacetate (DCFH-DA) was obtained from Beyotime Biotechnology Co., Ltd. (Shanghai, China). The detected probe for Fe^{2+} (FerroOrange) was bought from Dojindo (Japan). A lipid peroxidation probe (BODIPY 581/591 C11) was purchased from Thermo Fisher Scientific. The ferrostatin-1 (Fer-1), necrostatin-1 (Nec-1), 3-methyladenine (3-MA), and deferoxamine (DFO) were purchased from Sigma-Aldrich. Zinc protoporphyrin (ZnPP) was obtained from Aladdin Chemistry Co., Ltd. (Shanghai, China). Fetal bovine serum (FBS) and DMEM were obtained from Gibco BRL. All primers were purchased from Tsingke Biotechnology Co., Ltd. (Beijing, China). The HMOX1 and GPX4 antibodies were purchased from Abcom. The TrxR and Trx antibodies were obtained from Proteintech Group, Inc. All antibodies were used following the manufacturer's instructions. All aqueous solutions were prepared using ultrapure water (resistivity of 18.2 MΩ·cm). All air and moisture sensitive syntheses were carried out in flame-dried glassware with nitrogen atmosphere.

Syntheses of the intermediates: Compound **1**, compound **2** and pyridin-4-ylboronic acid as precursors were purchased from Zhengzhou Ruke Biotechnology Co., Ltd. $C_6F_5Au(tht)$ (tht = thiophene) was prepared according to the procedures described in the literature.[1]

Synthesis of compound 3: To a solution of compound **1** (963 mg, 1.30 mmol) and compound **2** (431 mg, 1.00 mmol) in THF/water (v/v: 5:1) mixture (42 mL) was added $Pd(PPh_3)_4$ (58 mg, 0.05 mmol) and K_2CO_3 (414 mg, 3.00 mmol). The mixture was stirred at 70 °C under N_2 for 4 h. And then the reaction was quenched by the addition of water (20 mL) and extracted with CH_2Cl_2 (3 × 20 mL). The combined organic extract was dried over anhydrous Na_2SO_4 and filtered. Solvent was removed by rotary evaporation. The product was purified by column chromatography (silica gel, CH_2Cl_2/hexane = 1:1, v/v) to obtain compound **3** as a light green solid. Yield: 463 mg (48 %). ^1H NMR (400 MHz, $CDCl_3$), δ (ppm): 8.62 (s, 1H), 8.62 (s, 1H), 7.41 (d, J = 8 Hz, 2H), 7.14 (d, J = 8 Hz, 4H), 7.00 (d, J = 4 Hz, 4H), 6.88 (d, J = 12 Hz, 2H), 3.83 (s, 6H, –OCH_3), 2.73 (d, J = 4 Hz, 2H), 2.60 (d, J = 4 Hz, 2H), 1.74 (m, 2H), 1.37-1.27 (m, 18H), 0.98-0.85 (m, 12H). ^{13}C NMR (100 MHz, $CDCl_3$), δ (ppm): 156.1, 150.4, 148.3,

146.0, 141.8, 140.6, 138.5, 137.1, 136.8, 134.8, 133.1, 129.8, 126.8, 126.3, 119.8, 116.8, 114.8, 113.5, 110.8, 55.5 (–O*C*H$_3$), 40.4, 40.0, 33.8, 32.6 (m), 28.8, 28.7, 25.8, 25.7, 23.2, 14.2 (d), 10.9 (d). HRMS (MALDI-TOF), m/z: calcd. for $C_{50}H_{54}BrN_5O_2S_4$: 963.2344; found: 963.2366.

Synthesis of TBTP: To a solution of compound **3** (963 mg, 1.00 mmol) and pyridin-4-ylboronic acid (160 mg, 1.30 mmol) in dioxane/THF/water (v/v/v: 3:2:1) mixture (42 mL) was added $Pd(PPh_3)_4$ (116 mg, 0.10 mmol) and K_2CO_3 (414 mg, 3.00 mmol). The mixture was stirred at 70 °C under N_2 for 12 h. And then the reaction was quenched by the addition of water (20 mL) and extracted with CH_2Cl_2 (3 × 20 mL). The combined organic extract was dried over anhydrous Na_2SO_4 and filtered. Solvent was removed by rotary evaporation. The product was purified by column chromatography (silica gel, CH_2Cl_2/MeOH = 20:1, v/v) to obtain compound TBTP as dark green solid. Yield: 635 mg (66 %). ^{1}H NMR (400 MHz, $CDCl_3$), δ (ppm): 8.85 (d, J = 8 Hz, 2H, pyridine-H), 8.68 (d, J = 4 Hz, 2H), 7.53 (d, J = 8 Hz, 2H, pyridine-H), 7.41 (d, J = 8 Hz, 2H), 7.13 (d, J = 12 Hz, 4H), 6.98 (d, J = 8 Hz, 2H), 6.87 (d, J = 8 Hz, 4H), 3.82 (s, 6H, –OC*H*$_3$), 2.81 (d, J = 8 Hz, 2H), 2.77 (d, J = 4 Hz, 2H), 1.75 (br, 2H), 1.26 (br, 18H), 0.88-0.84 (m, 12H). ^{13}C NMR (100 MHz, $CDCl_3$), δ (ppm): 156.1, 151.4, 151.0, 150.0, 148.5, 147.3, 146.4, 140.8 140.5, 138.7, 137.4, 137.0, 135.7, 129.8, 126.9, 126.1, 119.8, 114.8, 113.5, 110.8, 55.5 (–O*C*H$_3$), 40.5, 32.5 (m), 28.6 (d), 25.8, 23.1 (d), 14.2 (d), 10.9 (d). HRMS (MALDI-TOF), m/z: calcd. for $C_{55}H_{58}N_6O_2S_4$: 962.3504; found: 962.3515.

Synthesis of TBTP-Au: A mixture of C_6F_5Au(tht) (362 mg, 0.40 mmol) and TBTP (193 mg, 0.20 mmol) was stirred in CH_2Cl_2 (30 mL) for 48 h under N_2 at room temperature. After completion of the reaction, the solvent was evaporated. The crude product was recrystallized from CH_2Cl_2/iced diethyl ether to give a deep green solid in 93% yield (247 mg). ^{1}H NMR (400 MHz, THF-d$_8$), δ (ppm): 8.94 (d, J = 12 Hz, 2H), 8.72 (d, J = 4 Hz, 2H), 7.90 (d, J = 8 Hz, 2H), 7.43 (d, J = 4 Hz, 2H), 7.11 (d, J = 8 Hz, 4H), 6.98-6.88 (m, 6H), 3.98 (s, 6H), 2.93 (d, J = 8 Hz, 2H), 2.81 (d, J = 8 Hz, 2H), 1.73 (br, 2H), 1.42-1.29 (m, 18H), 0.92-0.86 (m, 12H). ^{13}C NMR (100 MHz, THF-*d*$_8$), δ (ppm): 157.9, 152.1, 151.9, 151.6, 149.6, 149.4, 148.5, 147.0, 143.8, 141.2, 140.7, 139.1, 138.7, 136.6, 136.5, 136.3, 136.2, 130.6, 128.1, 125.5, 120.2, 117.3, 115.8, 55.8 (*–OCH$_3$*), 41.6, 41.3, 34.9, 33.7 (d), 29.8, 29.7, 26.9, 24.2 (d), 14.8, 14.7, 11.4, 11.3. ^{19}F NMR (376 MHz, THF-*d*$_8$), δ (ppm): -116.0 (m), -159.2 (m), -162.8 (m). HRMS (MALDI-TOF), m/z: calcd. for $C_{61}H_{58}AuF_5N_6O_2S_4$: 1326.3090; found: 1326.3065.

Preparation of ApoE-TBTP-Au NPs: Firstly, the DSPE-TK-PEG$_{3400}$-ApoE was synthesized by Michael addition of ApoE-SH with DSPE-TK-PEG$_{3400}$-Mal in a nitrous atmosphere at

37 °C overnight. The DSPE-TK-PEG_{3400}-ApoE product was obtained after the resulting solution was further dialysis and freeze-dried. Next, 0.2 mL of tetrahydrofuran (THF) containing 1 mg of TBTP-Au, 1.2 mg of DSPE-TK-PEG_{3400}-ApoE, and 2.1 mg of DSPE-TK-PEG_{2000}, was poured into 10 mL of ultrapure water and removed the THF solvent at 70 °C for 30 min after sonicating for 1 min. Then, the ApoE-TBTP-Au NPs were acquired after centrifugation at 3700 rpm for 20 min by ultrafiltration tube with a molecular weight cutoff 100,000 Da. The ApoE-TBTP NPs also utilized the same preparation steps besides the TBTP instead of TBTP-Au. The TBTP-Au NPs also adopted the same procedure except for removing DSPE-TK-PEG_{3400}-ApoE and increasing the amount of DSPE-TK-PEG_{2000} to 2.6 mg.

Cell culture: The luciferase-tagged glioblastoma cells (U87-Luc) were utilized for *in vitro* tests and establishing the orthotopic glioma model. U87-Luc cells were cultured with Dulbecco's Modified Eagle's medium with 10% fetal bovine serum, 100 $U \cdot mL^{-1}$ penicillin and 100 $\mu g \cdot mL^{-1}$ streptomycin, and incubated at 37 °C with 5% CO_2.

***In vitro* anti-tumor studies:** CCK-8 assay was used to evaluate the cytotoxicity. U87-Luc cells were plated in 96-well plates (5×10^3 cells per well) for 24 h. The culture medium was individually replaced by a fresh medium containing various concentrations of nanoparticles for another 24 h. Subsequently, CCK-8 solution (10 µL) was added to each well for 1 h and measured the OD_{450} after washing three times with PBS. The cytotoxicity inhibition assays were carried out the same as the above procedures except adding ApoE-TBTP-Au NPs with various concentrations of inhibitors for 24 h, i.e., DFO (0, 400, 800 µM), ZnPP (0, 50, 100 µM), Fer-1 (0, 50, 100 µM), Nec-1 (12.5 µM), 3-MA (100 µM), and Z-DEVD-FMK (100 µM).

Target-enhanced penetration on 3D tumor spheroids model: To fabricate the U87 cells-based 3D tumor spheroids, we plated U87 cells (1×10^4 cells per well) in PrimeSurface 96-well plates and further incubated for 48 h. Then the U87 cells were respectively treated with target and nontarget NPs (100 µg/mL) for 4 h and washed with PBS three times. Last, the confocal laser scanning microscope (CLSM, Zeiss 980) imaging was used to monitor the permeability of NPs in every group.

RNA extraction and quantification: U87-Luc cells (2×10^5 cells per well) were seeded in 6-well plates and cultured overnight. Then the cells were respectively treated with ApoE-TBTP-Au NPs (100 µg/mL) for 24 h, PBS treatment served as a blank group. Total RNA was isolated from cells using RNAprep FastPure Tissue & Cell Kit (Tsingke Biotechnology Co., Ltd.) according to the manufacturer’s instructions. Total RNA (1 µg) was used as a template

to synthesize cDNA using PrimeScript RT Master Mix (Takara). PCR analysis was performed using a LightCycler 480II real-time detection system (Roche). The expression levels of target genes were normalized to that of GAPDH. The primers are shown as follows in Table S1.

Cell cycle test: First, the U87 cells without luciferase-tag (2×10^5 cells per well) were seeded in 6-well plates and cultured overnight. Then the medium was changed to an FBS-free medium and starved for 12 h. Subsequently, the cells were treated with PBS, ApoE-TBTP NPs (75 μg/mL) and ApoE-TBTP-Au NPs (75 μg/mL) for 24 h. The digested cell pellet suspensions were dropwise added to the 80% pre-cooled ethanol and fixed at -20 °C overnight. Last, the 10 μg of RNAase was added to PBS-cell suspension (1 μg/30 μL) and incubated at 37 °C for 30 min after centrifugation and suspended in PBS. The cell suspensions were stained with propidium iodide at room temperature for 30 min in dark and sent for flow cytometry analysis.

Tumor-bearing mouse model: All animal experiments were operated following the Guide Protocol of Laboratory Animals, approved by the Animal Care Committee of Henan University. BALB/c nude mice (female, 6-8 weeks) were purchased from SiPeiFu (SPF) (Beijing) Biotechnology Co., Ltd. U87-Luc cells (2×10^5) suspended in 5 μL of 0.9% NaCl were implanted into the left striatum of mice. Then the burr holes and scalp were respectively capped by bone wax and tissue glue. The tumor growth was monitored by bioluminescence using a fluorescence imaging system (IVIS, Lumina III; Caliper, USA).

Western blot assay: U87-Luc cells (2×10^5 cells per well) were seeded in 6-well plates and cultured overnight. The cells were treated with various nanoparticles for 24 h. Then the cells were harvested and lysed with 0.1 mL of RIPA buffer containing protease and phosphatase inhibitors. After that, the protein concentrations in all samples were quantified by a BCA kit. All were detached on a 10% SDS-PAGE and then transferred to a polyvinylidene difluoride membrane (PVDF membrane) that were blocked with 5% defatted milk for 1 h at room temperature. The membranes were incubated with the corresponding primary antibody ACTB (β-actin, 1:5000), GAPDH (1:5000), HMOX1 (1:1000), GPX4 (1:1000), Trx (1:5000), TrxR (1:5000), P53 (1:1000 dilution) at 4 °C overnight, following by another incubation of the secondary antibody (goat anti-rabbit IgG, goat anti-mouse IgG, 1:5000 dilution) at 25 °C for 1 h. After washing with TBST three times, the immunoreacted bands were recorded using ECL imaging observation by Super Signal ECL (Pierce). The protein analyses of brain tumor tissue were performed as same as the above procedures except the tissue samples were harvested after finishing the *in vivo* therapy process.

Pharmacokinetics and biodistribution: The pharmacokinetics curve was acquired on the healthy BALB/c mice after respectively injected with TBTP-Au NPs and ApoE-TBTP-Au NPs suspended in PBS buffer. The blood samples were harvested at predetermined time points to centrifuge at 3000 rpm for 15 min. After that, the supernatants were transferred to a 96-well plate and determined the OD value at 820 nm. The biodistribution assays were performed on the mice bearing orthotopic U87-Luc tumor at 6 h after intravenous injection of two NPs, the major tissues were homogenized in 0.2 mL of Triton X-100 (1%), lysed by 0.3 mL of DMF, and incubated at room temperature overnight. After that, the OD_{835} of the supernatants was measured after centrifuging at 15,000 rpm for 30 min. Routine blood parameters and blood biochemistry were performed on blood samples taken from healthy mice on day 10 after treatment with ApoE-TBTP-Au NPs (10 $mg{\cdot}kg^{-1}$) or PBS.

***In vivo* anticancer effect:** The orthotopic U87-Luc tumor-bearing mice were randomly divided into three groups: PBS, ApoE-TBTP NPs, and ApoE-TBTP-Au NPs groups. The corresponding samples were intravenously injected (dosage: 10 $mg{\cdot}kg^{-1}$) every three days for a total of four times. The tumor imaging and corresponding photon flux of ROI region in tumor were recorded using the Lumina IVIS III system. After finishing the whole therapy process, the major organs (heart, liver, spleen, lung, and kidney) of mice were retrieved for H&E staining, while the proteins in tumor tissues were homogenized in 0.2 mL of lysate at 4°C for 30 min. Then the supernatants were sent to the western blot analyses after centrifuging at 15,000 rpm for 30 min.

Statistical Analysis: All results are presented as means ± S.D. or mean ± S.E.M. according to at least three independent experiments. Statistical analysis was performed using GraphPad Prism (8.0). Two-tailed unpaired t-test was used for comparisons between two groups. The one-way analysis of variance (ANOVA) was used to compare multiple datasets. The survival curve was compared by the log-rank (Mantel-Cox) test. P value < 0.05 was considered statistically significant.

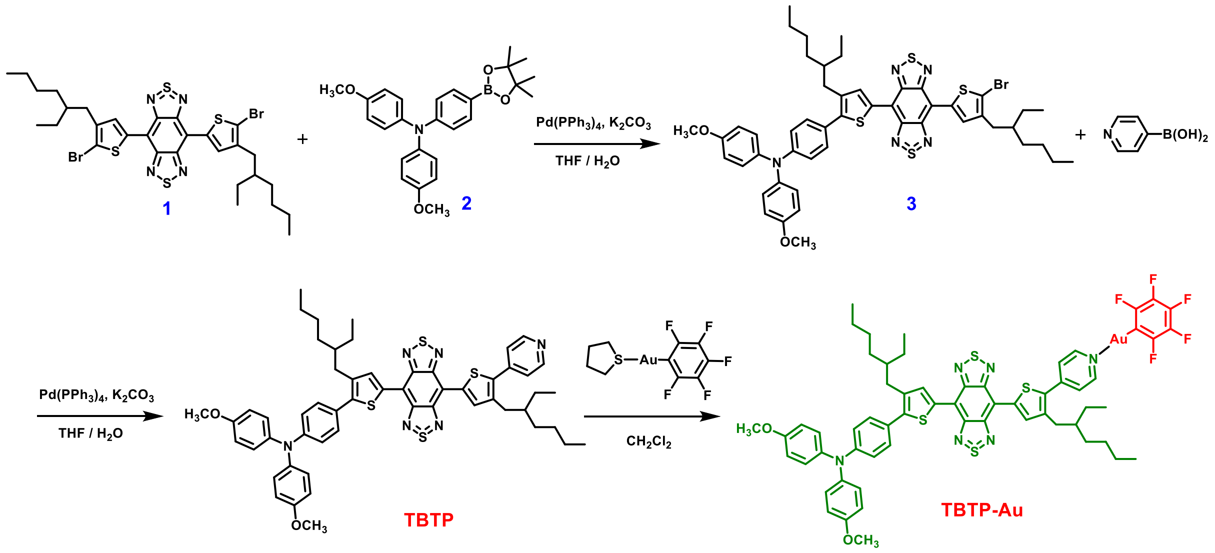

Figure S1. Synthetic route of the compound TBTP-Au.

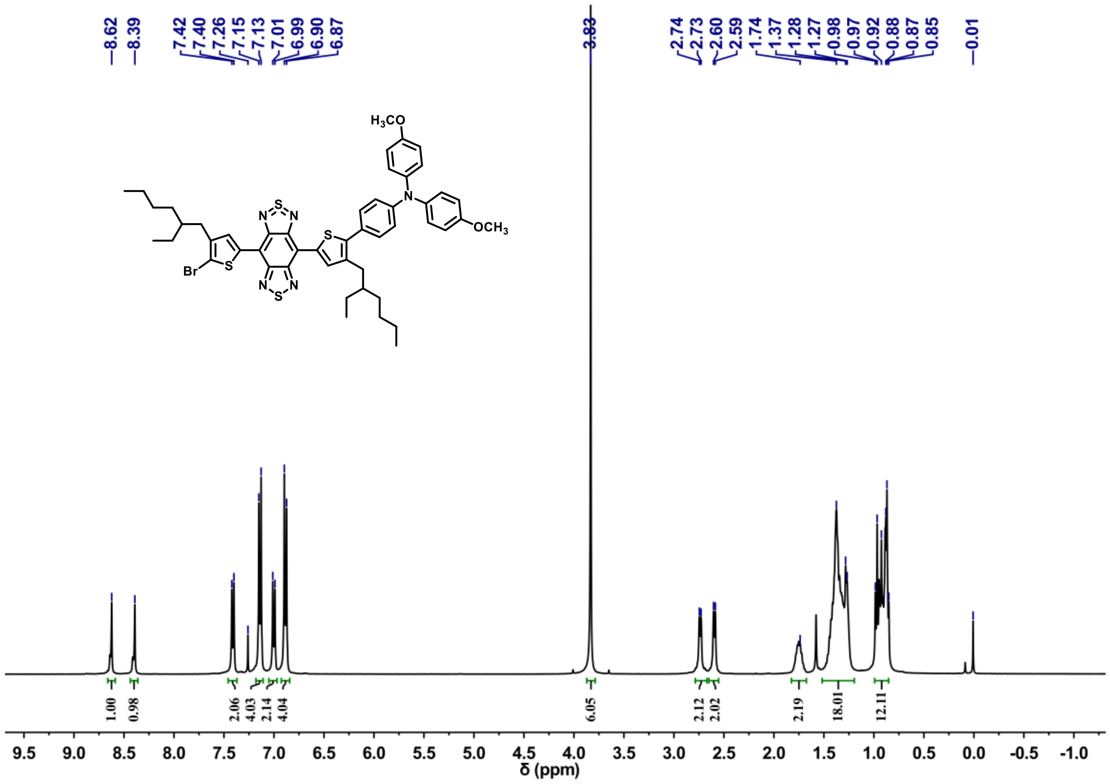

Figure S2. ^{1}H NMR spectrum of compound **3** in $CDCl_3$.

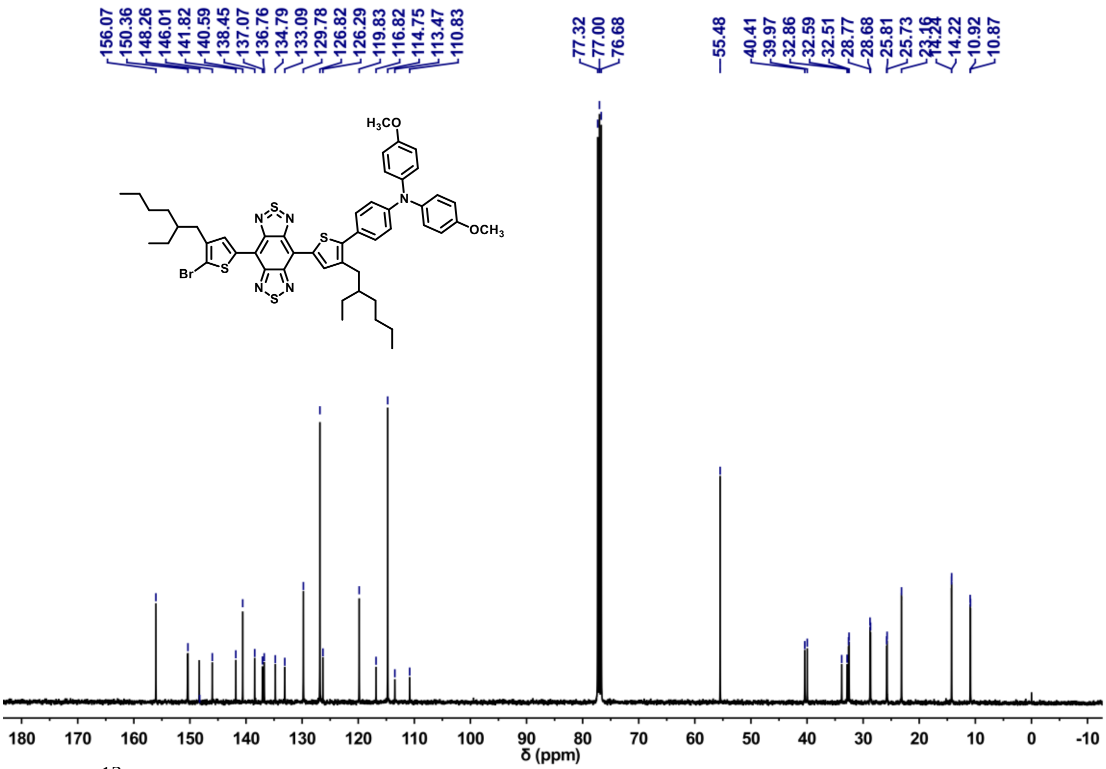

Figure S3. ^{13}C NMR spectrum of compound **3** in $CDCl_3$.

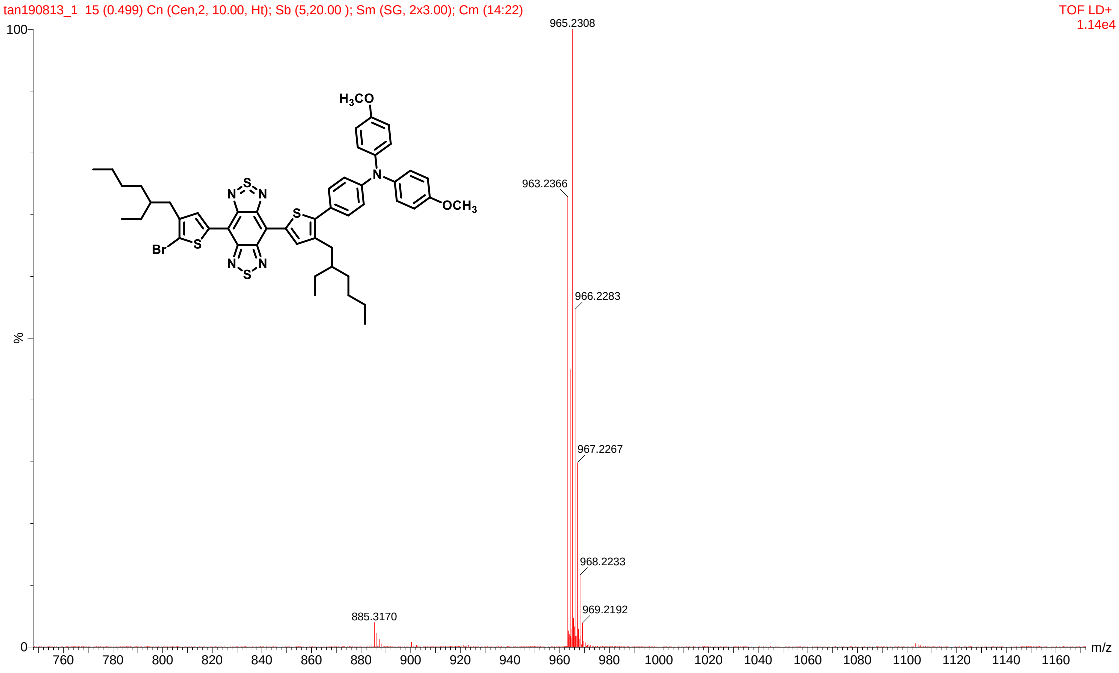

Figure S4. HR-MS spectrum of compound **3**.

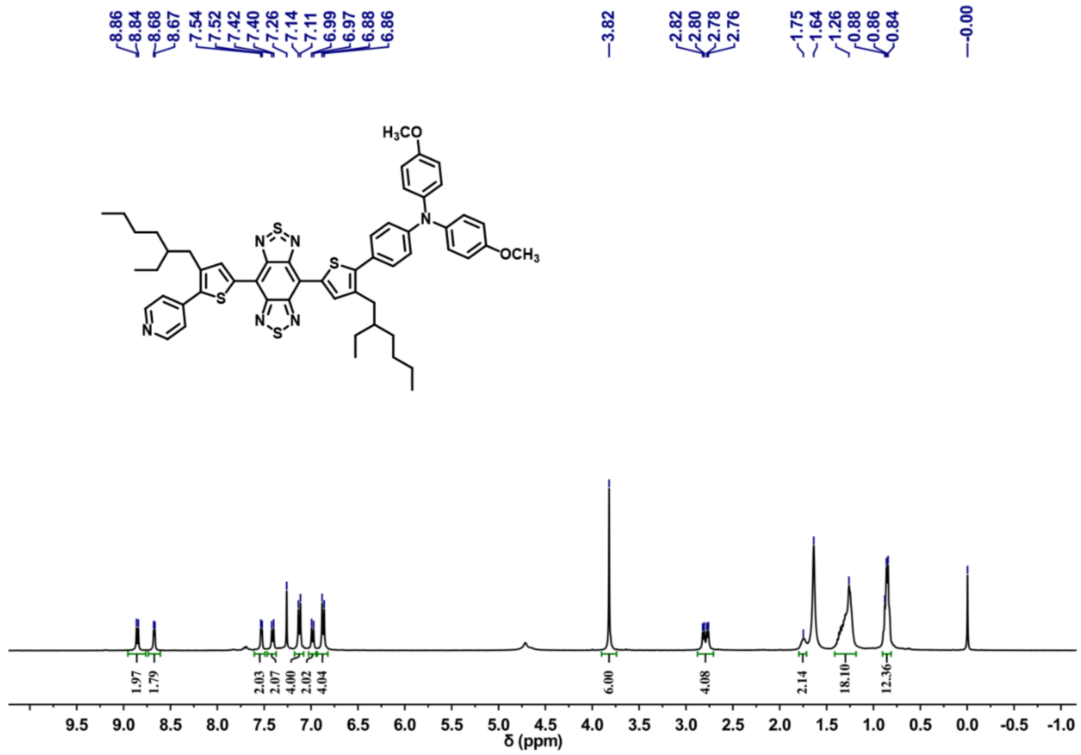

Figure S5. ^{1}H NMR spectrum of compound TBTP in $CDCl_3$.

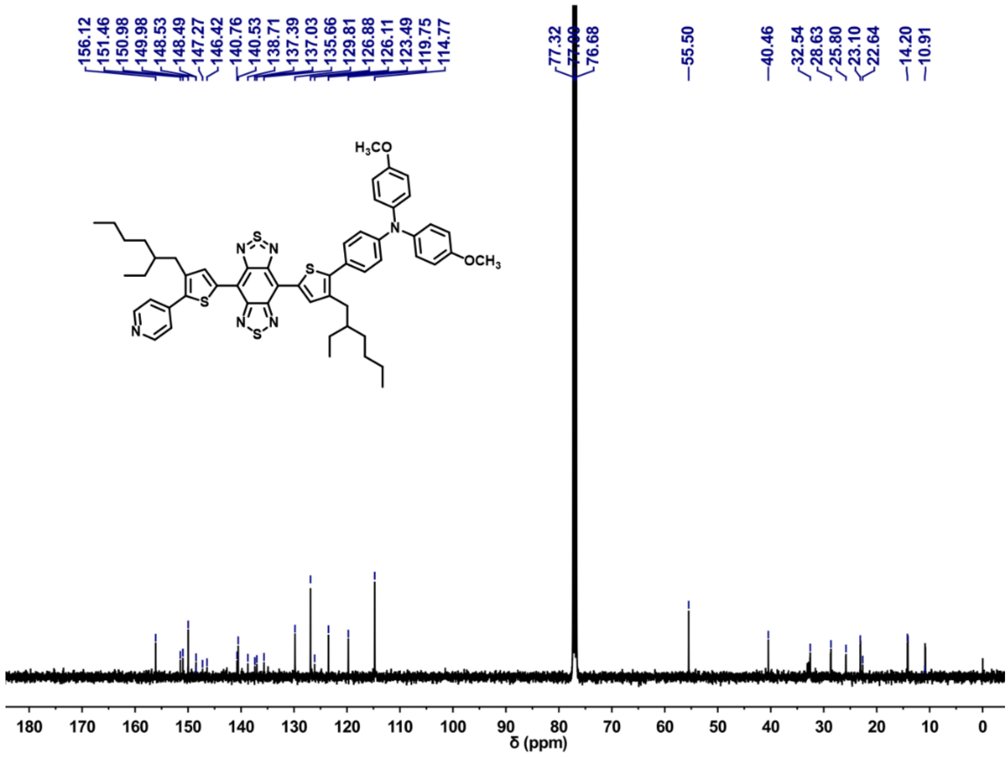

Figure S6. ^{13}C NMR spectrum of compound TBTP in $CDCl_3$.

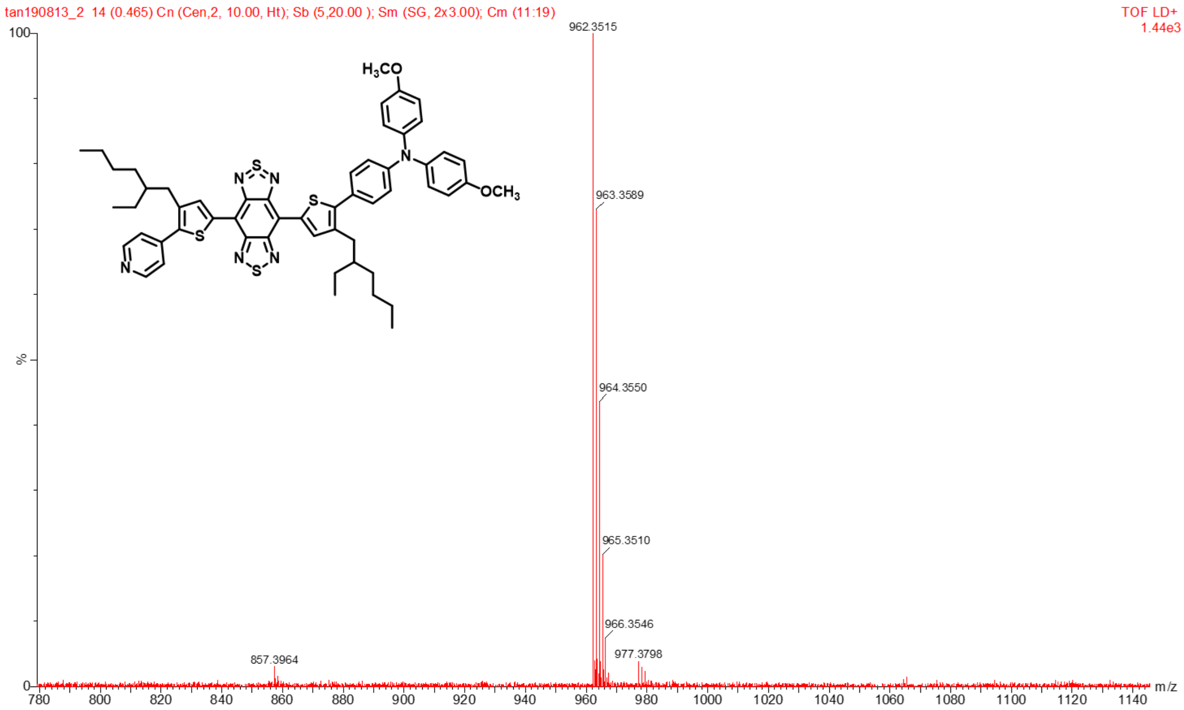

Figure S7. HR-MS spectrum of compound TBTP.

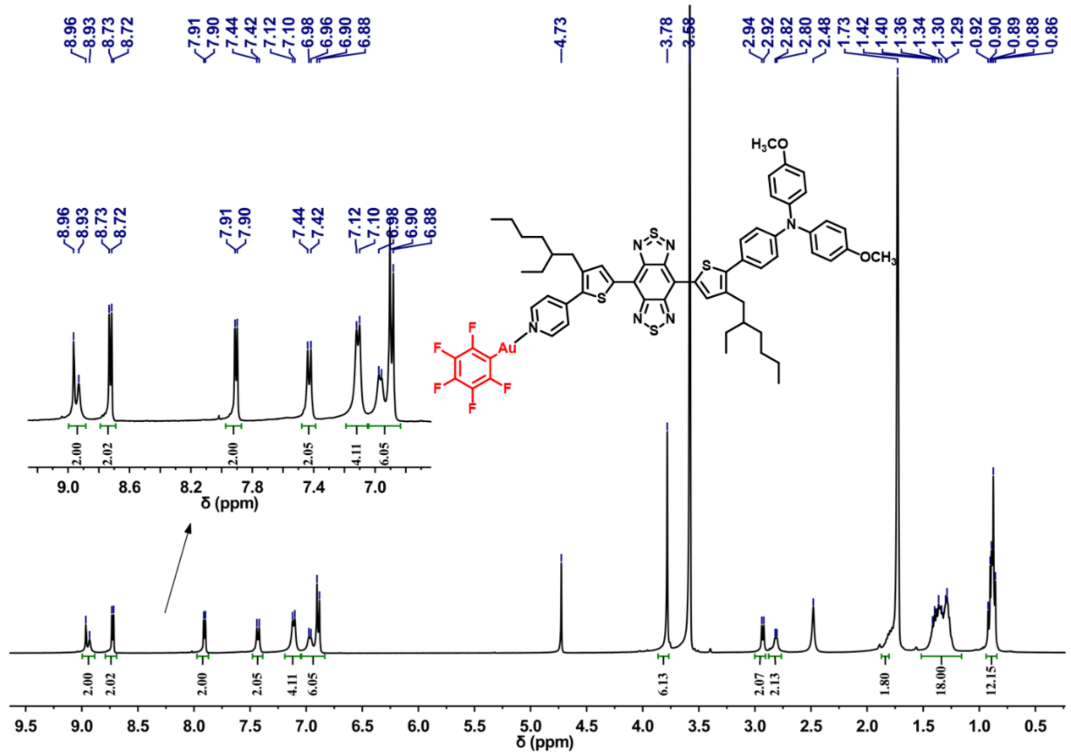

Figure S8. ^{1}H NMR spectrum of TBTP-Au in THF-d_8.

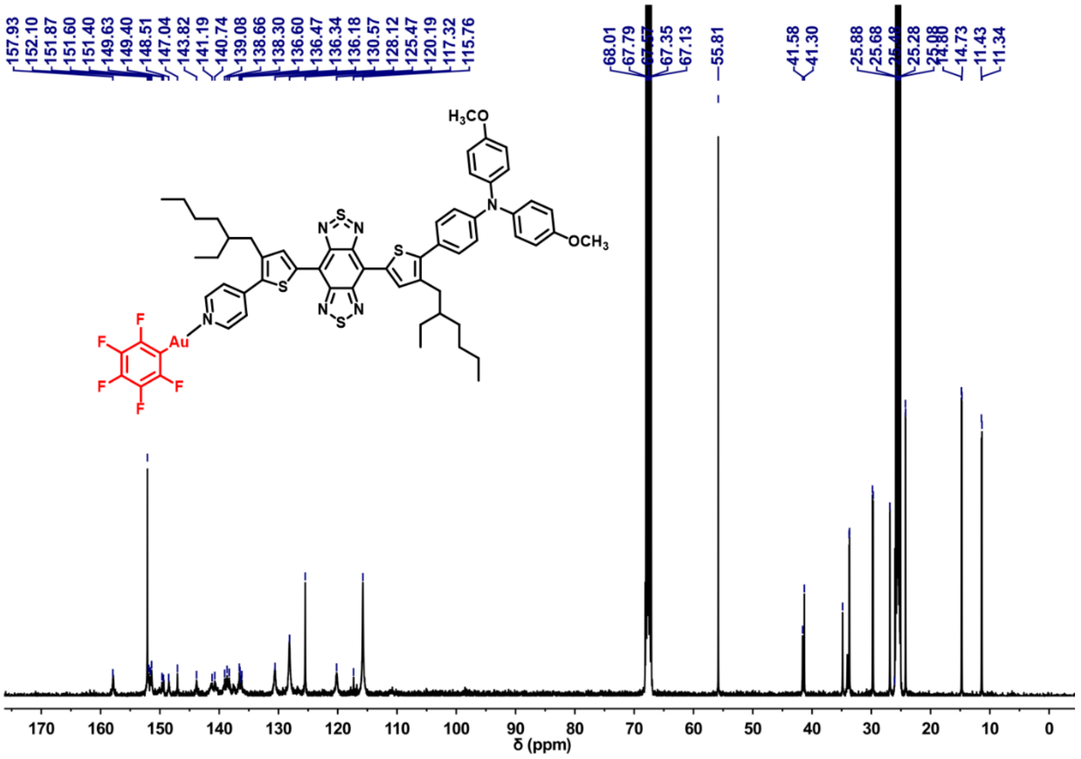

Figure S9. ^{13}C NMR spectrum of TBTP-Au in THF-d_8.

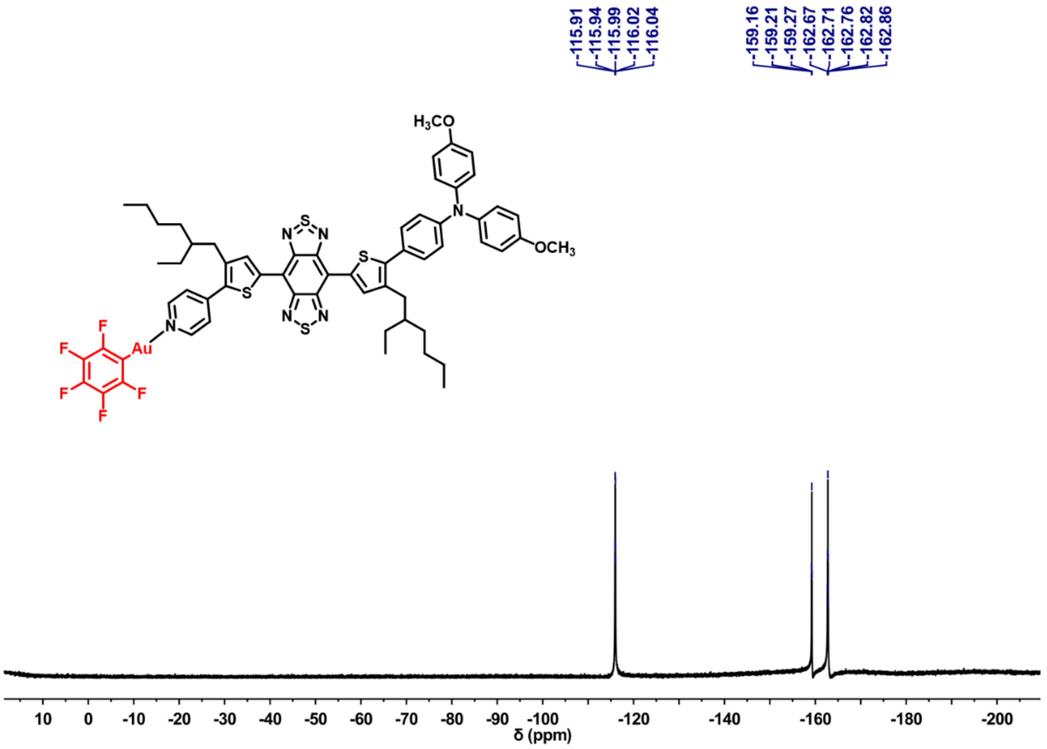

Figure S10. ^{19}F NMR spectrum of TBTP-Au in THF-d_8.

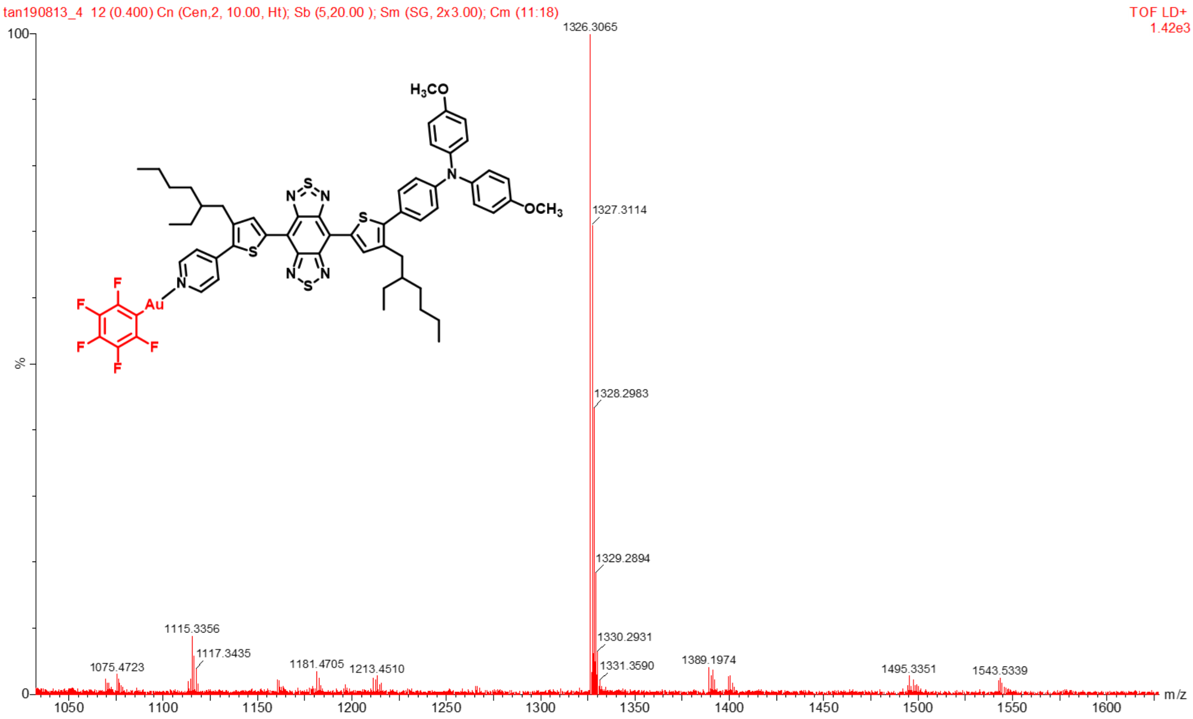

Figure S11. HR-MS spectrum of TBTP-Au.

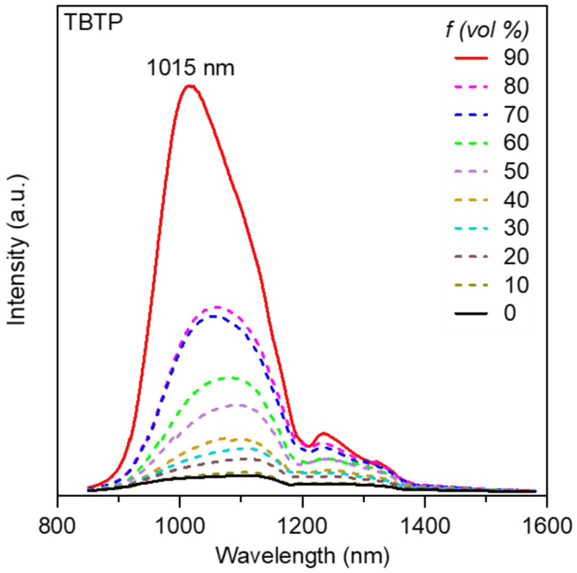

Figure S12. Photoluminescence spectra of TBTP in THF/*n*-hexane mixture with different fractions (*f*) of *n*-hexane (excitation: 808 nm).

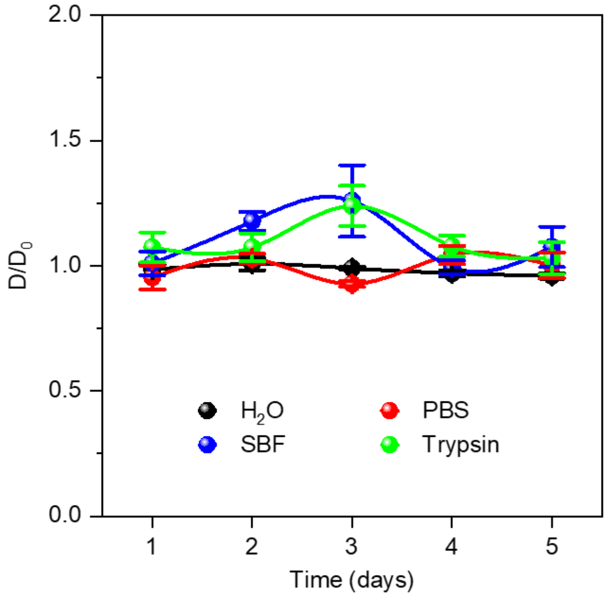

Figure S13. Diameter ratio (D/D_0) variations of TBTP-Au NPs in different media, D_0 was the initial diameter.

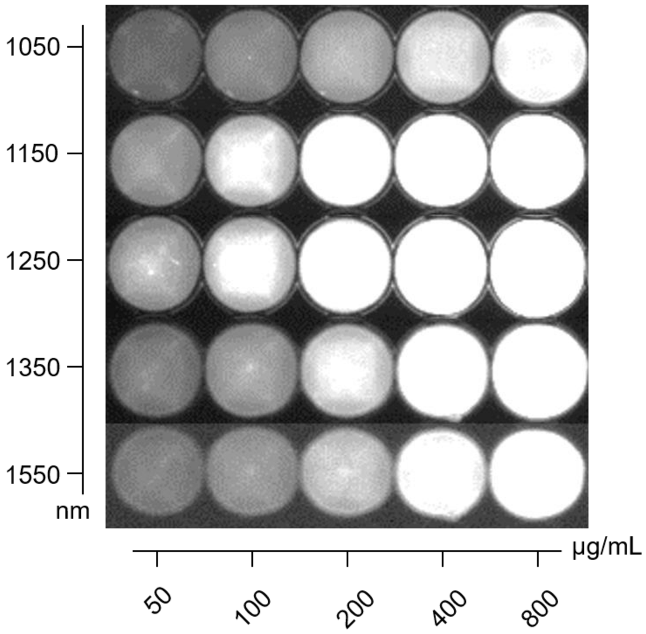

Figure S14. NIR-II imaging of various concentrations of TBTP-Au NPs aqueous solution under different bandpass filters.

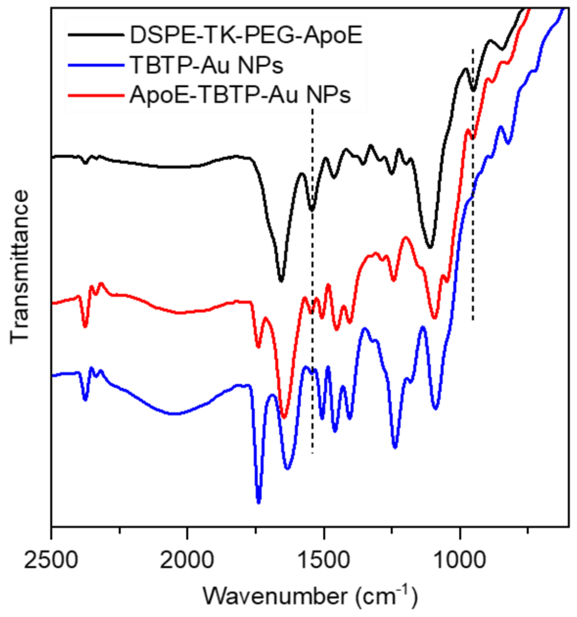

Figure S15. Fourier-transform infrared spectroscopy (FTIR) spectra of various samples.

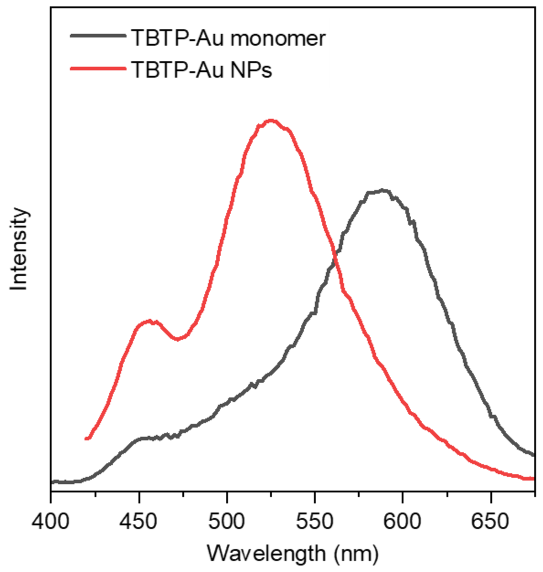

Figure S16. Visible PL spectra of TBTP-Au NPs dispersed in deionized water and TBTP-Au monomer dissolved in tetrahydrofuran solvent.

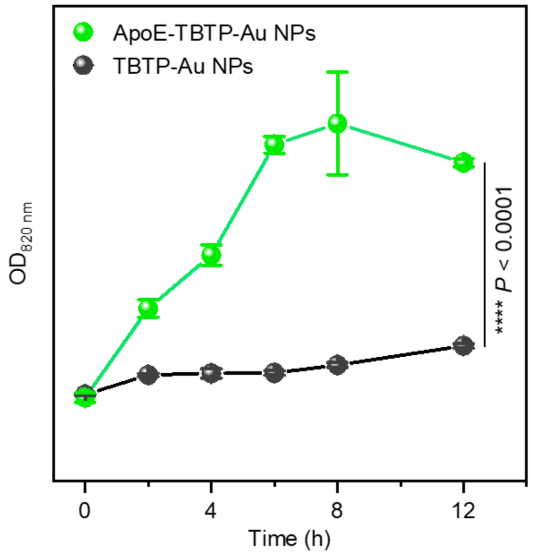

Figure S17. The uptake was quantified through the optical value at 820 nm of U87 cells incubated with nontarget and target NPs (100 μg/mL) for various times. Data are presented as means ± standard error of the mean (SEM).

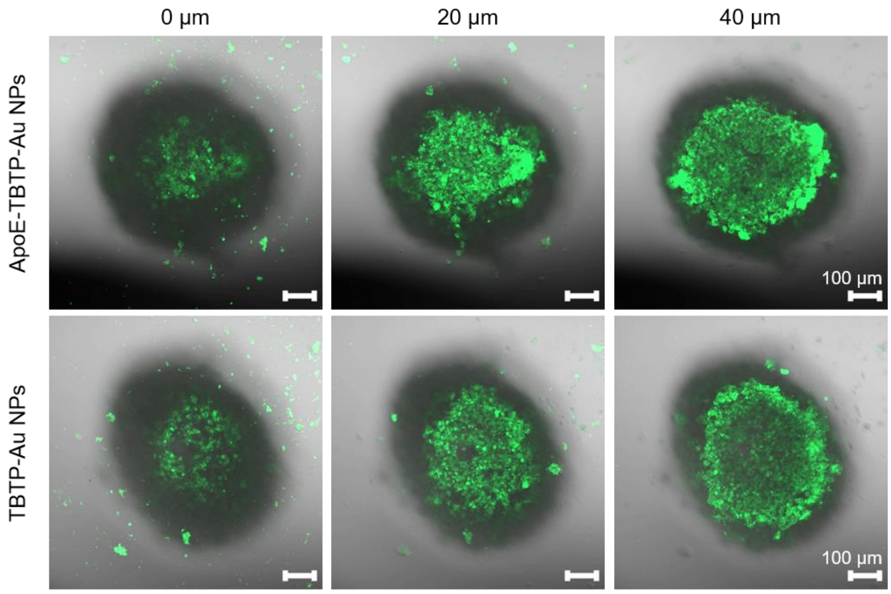

Figure S18. Confocal images of U87 tumor spheroids incubated with nontarget and target NPs (100 μg/mL) for various depths. Scale bars: 100 μm.

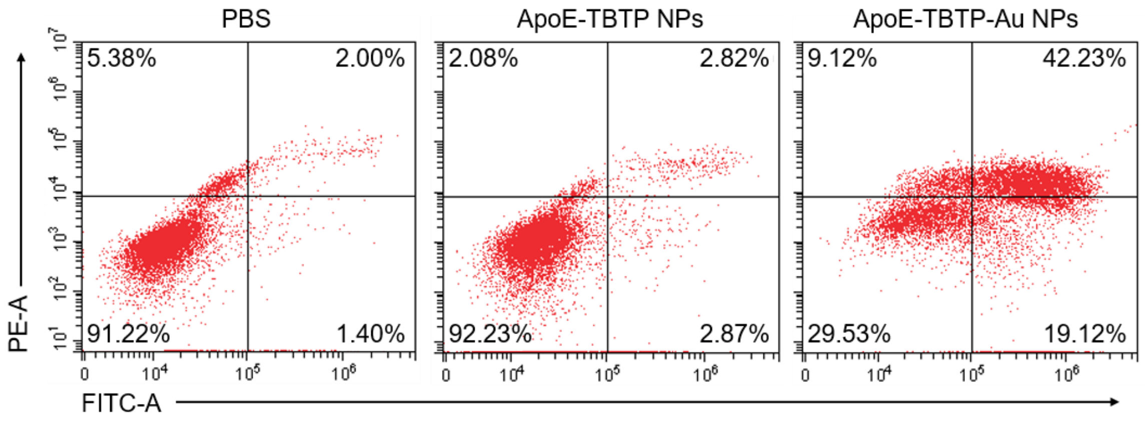

Figure S19. Flow cytometry analyses. The U87 cells were treated respectively with ApoE-TBTP NPs (100 μg/mL) and ApoE-TBTP-Au NPs (100 μg/mL) for 24 h.

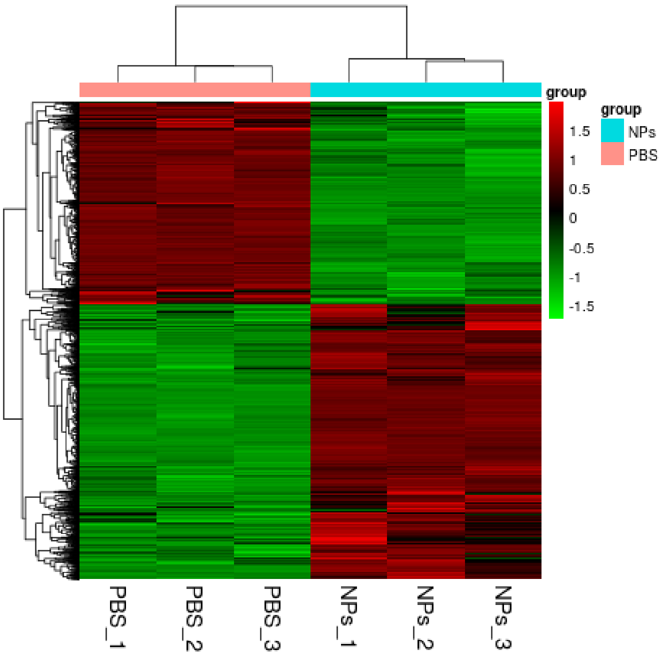

Figure S20. Heat map of identified DEGs in U87 cells treated with ApoE-TBTP-Au NPs for 24 h compared with PBS group (n = 3). The gene expression matrix was normalized at row direction using Z-score (range from -1.5 to +1.5). Clustering analysis is conducted in column and row direction using pheatmap software (R package).

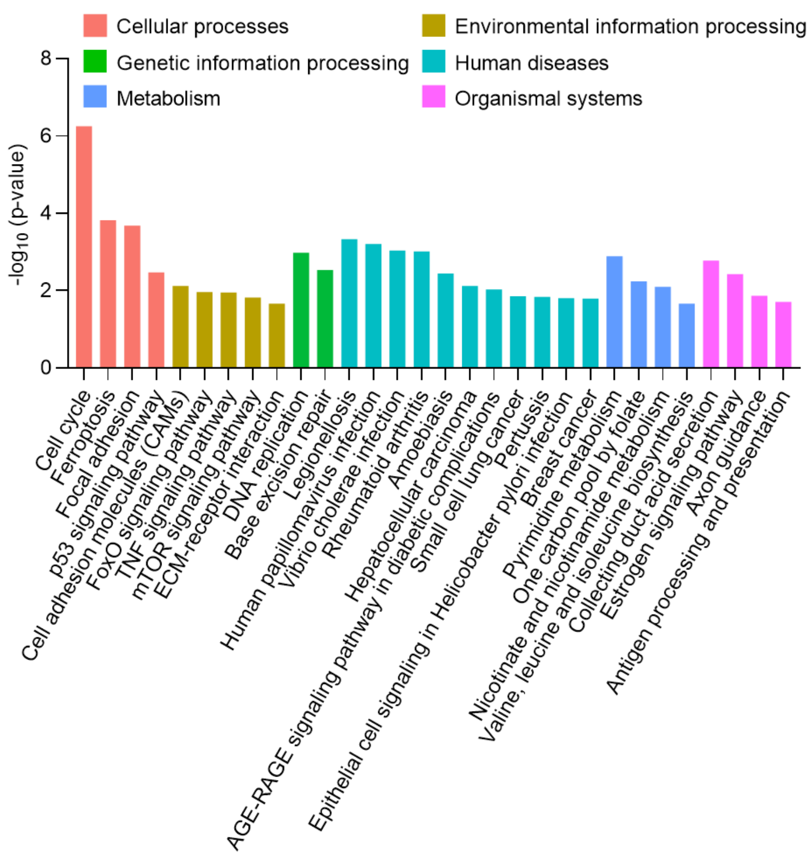

Figure S21. Kyoto Encyclopedia of Genes and Genomes (KEGG) analysis. Histogram profiles of enriched terms are clustered by different biological processes.

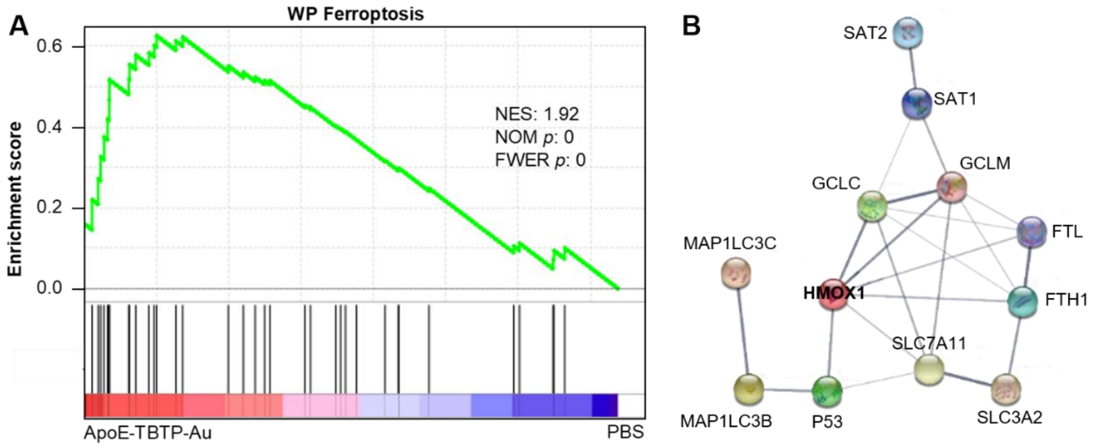

Figure S22. A) Gene Set Enrichment Analysis (GSEA) plot between ApoE-TBTP-Au NPs and PBS groups. B) Protein-protein interaction (PPI) network analysis reveals that the major proteins influenced the ferroptosis pathway.

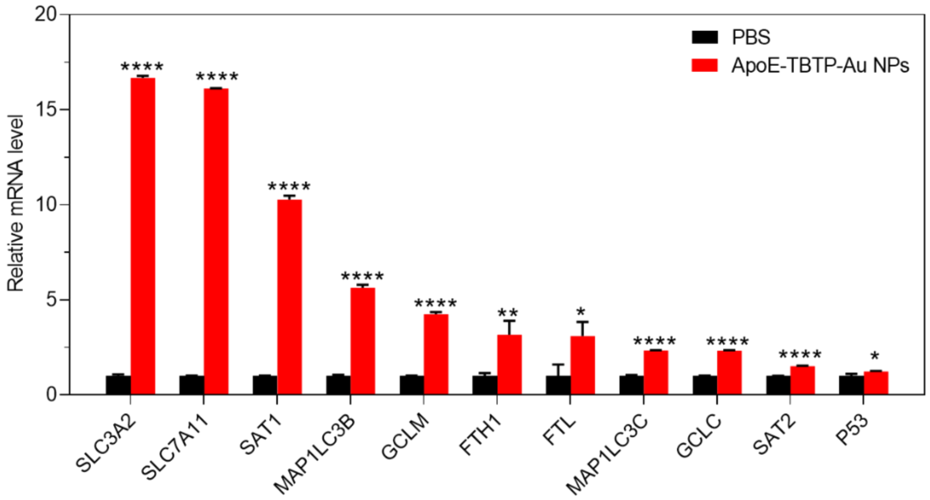

Figure S23. Relative mRNA expression of U87 cells respectively treated with ApoE-TBTP-Au NPs and PBS (n = 3). Unpaired student′s *t*-testing, *, $p < 0.05$, **, $p < 0.01$, ****, $p < 0.0001$.

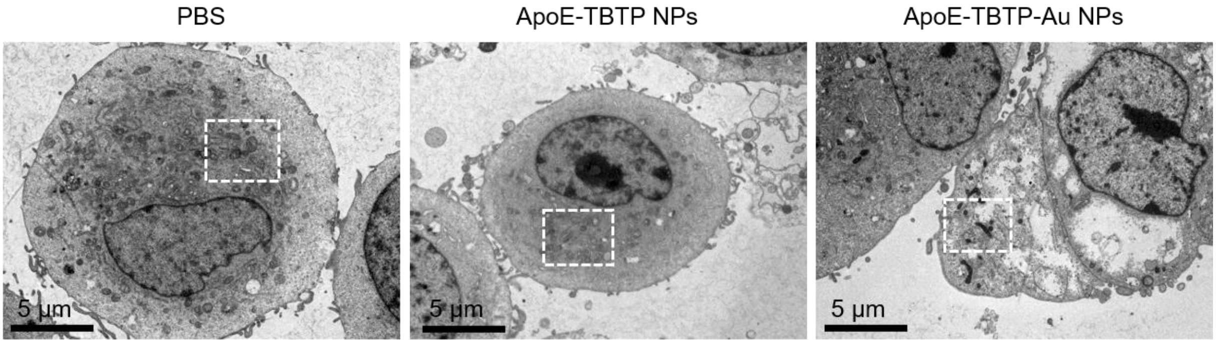

Figure S24. Bio-TEM observation of U87 cells respectively treated by PBS, ApoE-TBTP NPs (100 μg/mL), and ApoE-TBTP-Au NPs (100 μg/mL) for 24 h.

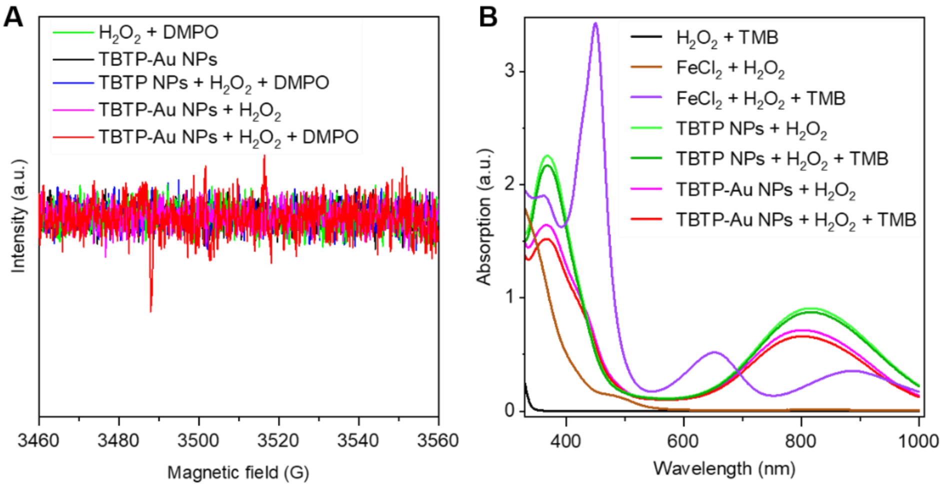

Figure S25. A) Electron paramagnetic resonance (EPR) spectra of various samples. The 5,5-dimethyl-1-pyrroline N-oxide (DMPO) was used to capture the free radical. B) The

absorption changes of various solutions under 100 μM of H_2O_2. The 3,3′,5,5′-tetramethylbenzidine (TMB, 20 μg/mL) was used as the visualizing reagent for radical.

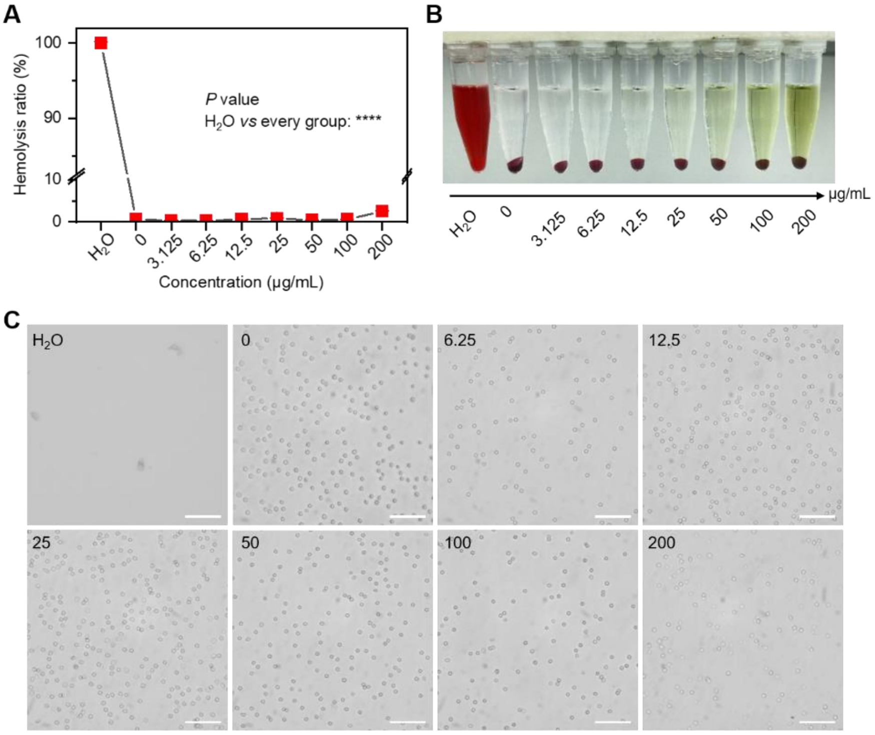

Figure S26. A) Hemolysis ratio test of erythrocyte cells treated by H_2O, PBS, and various concentrations of ApoE-TBTP-Au NPs in PBS. Notably, the absorption of supernate was used after subtracting the background value of PBS or NPs. B) The corresponding photos of the centrifuged solution. C) The morphology of erythrocyte cells (Units: μg/mL). Scale bars: 50 μm. Data are presented as mean ± standard deviation (SD). $^{****}P < 0.0001$.

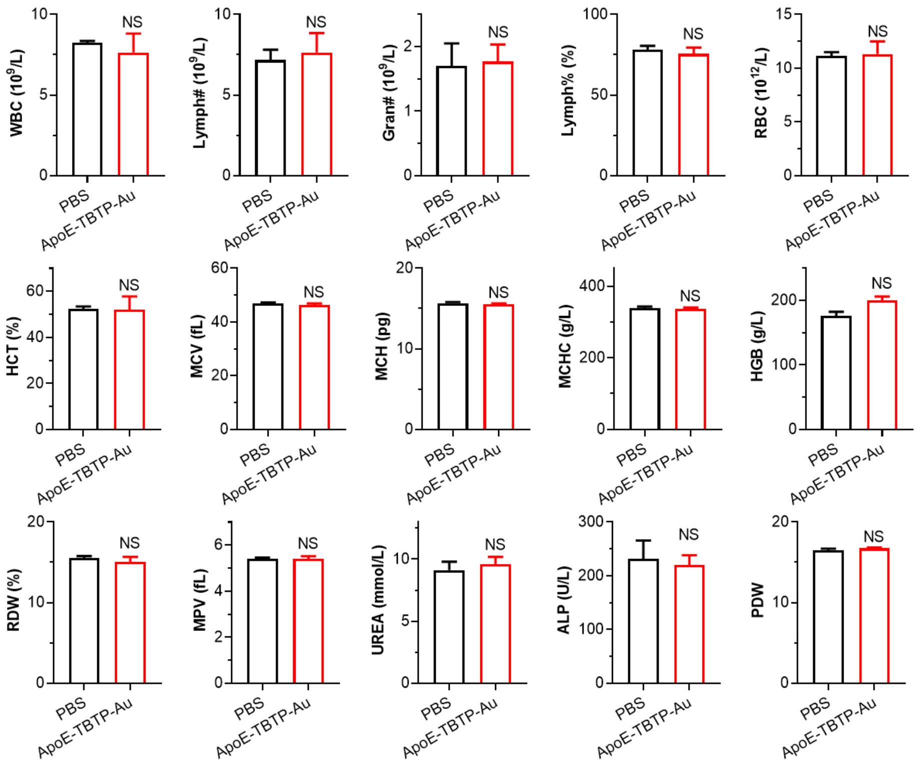

Figure S27. Bio-safety assessment. Routine blood parameters and blood biochemistry tests on healthy mice with PBS and ApoE-TBTP-Au NPs treatments. Data are presented as means ± standard error of the mean (SEM). NS, no significance.

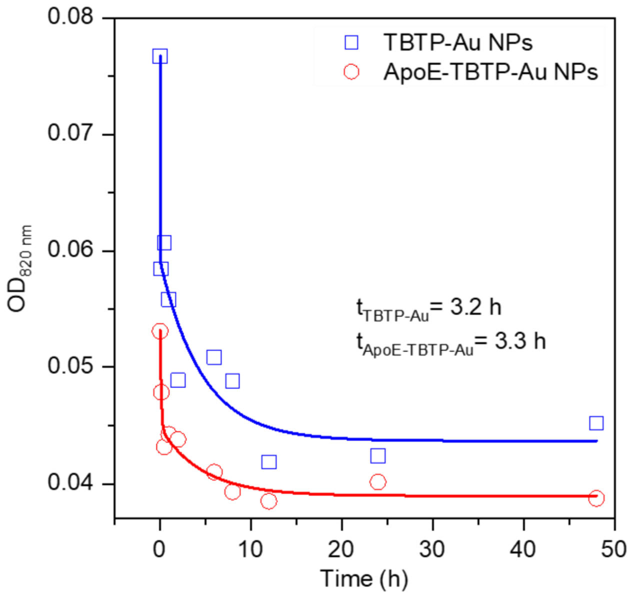

Figure S28. *In vivo* pharmacokinetic study of healthy mice that were *i.v.* injected with nontarget and target NPs (dosage: 10 mg/kg).

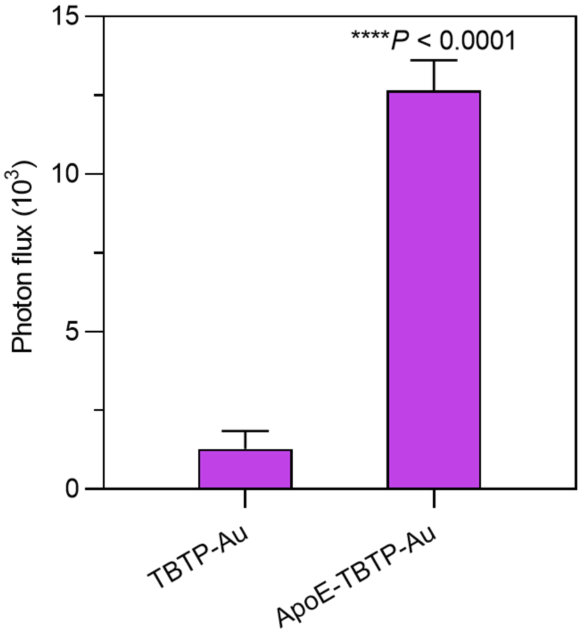

Figure S29. The quantitative biodistribution of two NPs in brain through the photon flux value of NIR-II images at 1350 nm (n = 3). Data are presented as mean ± standard deviation (SD). ****P < 0.0001.

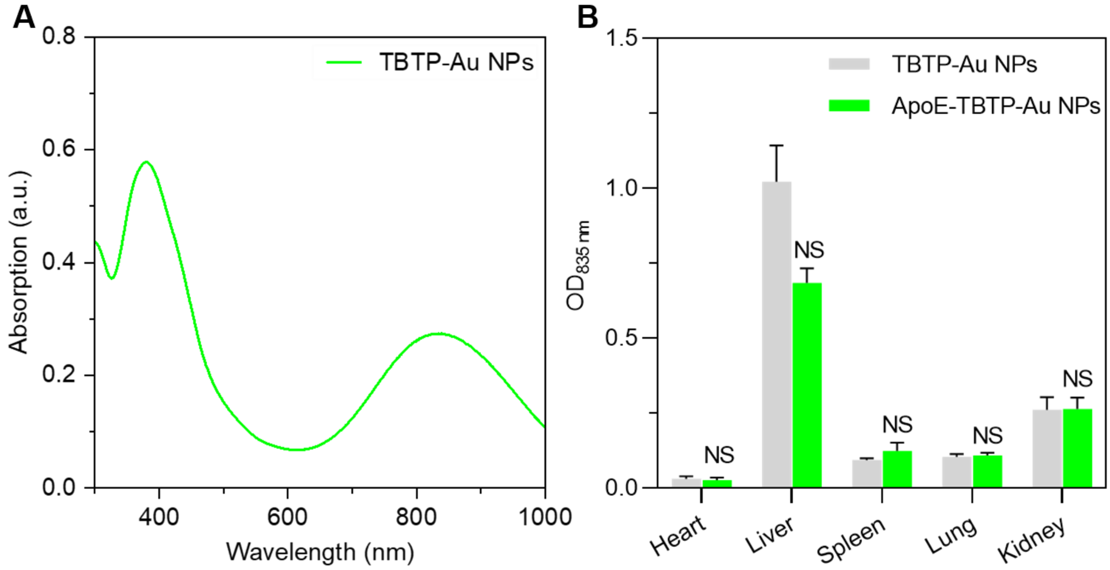

Figure S30. Quantification of the distribution of NPs in main organs. A) Absorption spectra of TBTP-Au NPs dissolved in the mixed solvent of DMF with Triton X-100. B) The corresponding optical density (OD) values at 835 nm of NPs distributed in various tissues. These tissues were homogenized and dissolved in the above mixed solvent. NS, no significance. Data are presented as mean ± standard deviation (SD). NS, no significance.

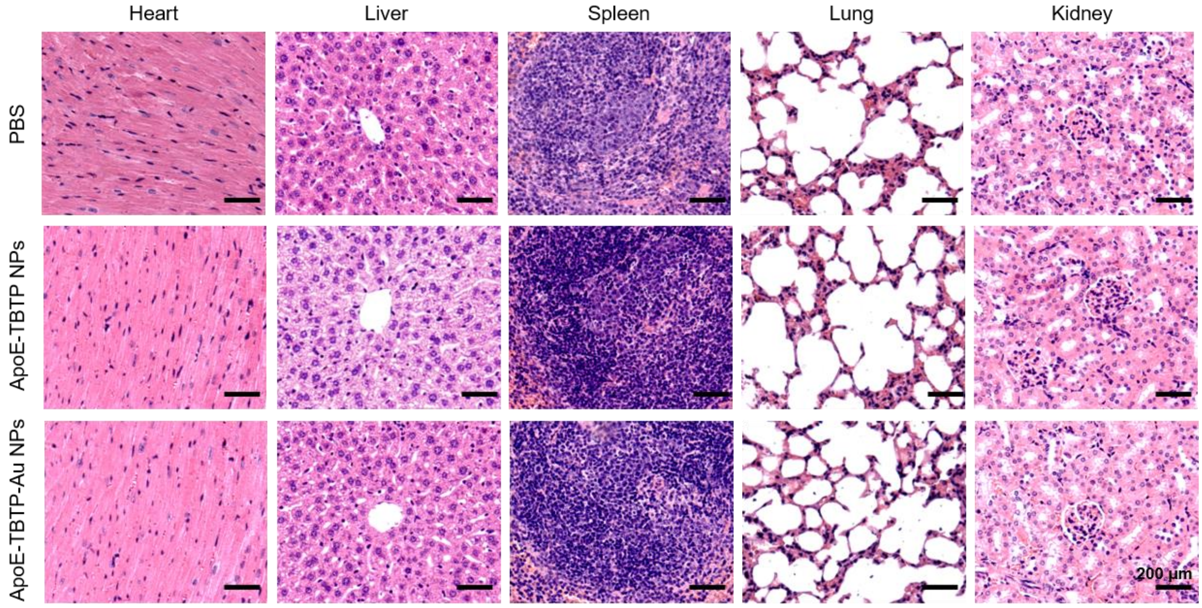

Figure S31. Hematoxylin-eosin (H&E) staining observation from main organs of mice treated with PBS, ApoE-TBTP NPs, and ApoE-TBTP-Au NPs. Scale bars: 200 μm.

Table S1. The PCR primers and gene sequences in this study are shown as follows:

Gene	Forward primer (5′-3′)	Reverse primer (5′-3′)
	Human genes	
HMOX1	GAGCCTGGAAGACACCCTAA	GGCCGTGTCAACAAGGATAC
SLC7A11	TGGAGTCCCTGCGTATTATC	ATTGCCAAGATCTCAAGTCC
GCLC	AACTTCATTTCCCAGTACCTT	TTTCTCCTCTCCTATGTCGG
GCLM	GAGGAGGAGTTTCCAGATGT	CCATGTCAACTGCACTTCTA
SAT1	AAGATGGTTTTGGAGAGCAC	CAATGCTGTGTCCTTCCG
SLC3A2	TTACCTGAGCTCTCTGAAGG	CGACATCATCCTTCTGGTTC
MAP1LC3B	CTGAGTCTTCTCTTCAGGTTC	TTTACAGTCAGGGCCGTTTT
FTL	CGTCAGAATTATTCCACCGA	CAGAGAGAGGTAGGTGTAGG
FTH1	AGAACTACCACCAGGACTCAG	GTAAACGTAGGAGGCGTAGA
SAT2	CTGATTCGGGTGAAGACTG	AGGGCTTCTTCACTGATCTT
MAP1LC3C	GAATGAGTTAGGTTCCCGGTT	CACTACCACCGGGATTTTG
GAPDH	ACCCAGAAGACTGTGGATGG	TCAGCTCAGGGATGACCTTG
P53	AAGCTGTCCCTCACTGTTGA	TGCACTCTGTGAGGTAGGTG

Gene	shRNA sequence
HMOX1-shRNA-1	CCGG-ACAGTTGCTGTAGGGCTTTAT-CTCGAG-ATAAAGCCCTACAGCAACTGT-TTTTTG
HMOX1-shRNA-2	CCGG-CATCCAGGCAATGGCCTAAAC-CTCGAG-GTTTAGGCCATTGCCTGGATG-TTTTTG

Reference

[1] R. Uson, A. Laguna, M. Laguna, D. A. Briggs, H. H. Murray, J. P. Fackler Jr. *Inorg. Synth.* **1989**, *26*, 85.
